# Is the relative thickness of ammonoid septa influenced by ocean acidification, phylogenetic relationships and palaeogeographic position?

**DOI:** 10.1186/s13358-022-00246-2

**Published:** 2022-04-18

**Authors:** Céline Weber, Michael Hautmann, Amane Tajika, Christian Klug

**Affiliations:** 1 Paläontologisches Institut Und Museum, Universität Zürich, Karl-Schmid-Strasse 4, 8006 Zurich, Switzerland; 2 Division of Paleontology (Invertebrates), American Museum of Natural History, Central Park West 79th Street, New York, NY 10024 USA; 3 University Museum, University of Tokyo, 7-3-1 Hongo, Tokyo, 113-0033 Japan

**Keywords:** Ammonoids, Septal thickness, End-Triassic mass extinction, Mesozoic, Ocean acidification, Calcifying organism, Atmospheric CO_2_, Seawater pH

## Abstract

The impact of increasing atmospheric CO_2_ and the resulting decreasing pH of seawater are in the focus of current environmental research. These factors cause problems for marine calcifiers such as reduced calcification rates and the dissolution of calcareous skeletons. While the impact on recent organisms is well established, little is known about long-term evolutionary consequences. Here, we assessed whether ammonoids reacted to environmental change by changing septal thickness. We measured the septal thickness of ammonoid phragmocones through ontogeny in order to test the hypothesis that atmospheric *p*CO_2_, seawater pH and other factors affected aragonite biomineralisation in ammonoids. Particularly, we studied septal thickness of ammonoids before and after the ocean acidification event in the latest Triassic until the Early Cretaceous. Early Jurassic ammonoid lineages had thinner septa relative to diameter than their Late Triassic relatives, which we tentatively interpret as consequence of a positive selection for reduced shell material as an evolutionary response to this ocean acidification event. This response was preserved within several lineages among the Early Jurassic descendants of these ammonoids. By contrast, we did not find a significant correlation between septal thickness and long-term atmospheric *p*CO_2_ or seawater pH, but we discovered a correlation with palaeolatitude.

**Supplementary Information:**

The online version contains supplementary material available at 10.1186/s13358-022-00246-2.

## Introduction

The long-term effects of environmental change on organisms spanning more than some millennia can only be studied using the fossil record. Palaeontological data, however, may lack precise age control that is required for that purpose, but quickly evolving taxa that are commonly used as biostratigraphic index fossils allow the determination of their geologic age from their taxonomic identity. Such a group is the ammonoids, extinct cephalopods that rapidly evolved after their origin in the Devonian oceans over 407 million years ago (Becker et al., 2020). They are widely used index fossils and usually record their ontogeny in their aragonitic shell. They also represent excellent model organisms to examine the effect of oceanic environmental change from their origin in the Early Devonian (Becker et al., 2019, 2020; Klug et al., 2015) to their demise at the end of the Cretaceous (De Baets et al., 2016; Landman et al., 2015; Tajika et al., 2018, 2020). During this interval, ammonoids experienced dramatic diversity-fluctuations due to several major extinction events (end-Devonian, end-Permian and end-Triassic; Arkhipkin & Laptikhovsky, 2012; Veron, 2008) as well as numerous smaller scale extinctions (Becker et al., 2020; Korn et al., 2015).

The ammonoid conch consists of aragonite, which is the metastable polymorph of calcium carbonate and 1.5 times more soluble than calcite (Morse & Mackenzie, 1990). The conch is subdivided into the body chamber, which contains the soft tissue, and the phragmocone that consists of individual chambers separated by septa, which are connected by the partially organic siphuncle. Although the septa are not in direct contact with seawater, the formation of septa was likely still affected by differences in seawater chemistry (Immenhauser et al., 2016). Biocalcification is an active, physiological process that requires significant amounts of energy for the manipulation of seawater to form a calcifying fluid (Palmer, 1992). More precisely, every µmol of CaCO_3_ produced by biocalcifiers requires the removal of two µmol protons (H^+^ ions from the hydrogencarbonate ions HCO_3_^−^) from the calcifying fluid, and the expenditure of 1 µmol of “energy” or ATP (Cohen & Holcomb, 2009). A biocalcifier building a normal skeleton under elevated CO_2_ conditions would have to divert more energy to pumping protons from the calcifying fluid than a biocalcifier building a normal skeleton under ambient CO_2_ conditions (Cohen & Holcomb, 2009). For this reason, ocean acidification also affects the formation of calcareous shells that are not in direct contact with seawater, and thinner septa might be formed if the organism cannot provide sufficient energy for maintaining ‘normal’ thickness under reduced seawater pH. Aragonite biomineralisation requires more modification of seawater and hence more energy than calcite biomineralisation because seawater is less saturated for aragonite than for calcite (e.g., Morse & Mackenzie, 1990); thus, aragonitic biocalcifiers are more susceptible to ocean acidification than calcitic biocalcifiers (Hautmann, 2006; Hautmann et al., 2008). However, there are exceptions. Langer et al. (2014) studied the response of the patellogastropod limpet *Patella caerulea* to reduced seawater pH near a CO_2_ vent at Ischia (Italy) and found that the animal compensated for the dissolution of its calcitic outer shell layer by the secretion of additional aragonitic shell material in the inner shell layer. Similarly, juveniles of the cephalopod mollusc *Sepia officinalis* were observed to maintain calcification under ~ 4000 and ~ 6000 ppm CO_2_ and to grow at the same rate with the same gross growth efficiency as did control animals, including the formation of their calcified cuttlebone (Gutowska et al., 2010). These examples show that no general prediction on the effects of reduced seawater pH on calcifying organisms can be made and that more data for different groups of organisms are required, which is the aim of this study.

Some researchers demonstrated the impact of fluctuations in abiotic factors such as pH and CO_2_ on the formation of carbonate skeletons in many marine organisms (corals, coccolithophorids echinoderms, bivalves and gastropods; Gazeau et al., 2007), but the impact of ocean acidification on ammonoid septa remained unknown. In addition, although several studies provided indirect evidence that environmental stress influenced the growth of the ammonoid phragmocone, partially due to their moderately high metabolism (e.g., Kraft et al., 2008; Tajika et al., 2015, 2020), there is no data available on how septal thickness covaries with factors such as seawater pH, latitude, climate, water depth, or systematic relationship.

The long-term effects of climate change and decreasing pH of sea water (commonly dubbed ocean acidification) on marine organisms have important actualistic implications. The current rise of anthropogenic atmospheric partial pressure of carbon dioxide (*p*CO_2_) and the resulting uptake of CO_2_ by the oceans causes a decrease of pH and the carbonate saturation state (Zeebe, 2012). This CO_2_-induced ocean acidification and its resulting impact on the marine environment is currently subject of intense research. Many studies focus on the present and near future (e.g., Gattuso et al., 1998; Langdon & Atkinson, 2005; Langdon et al., 2000; Rodolfo-Metalpa et al., 2011) while some researchers also examined the geological past (Hautmann et al., 2008; Knoll et al., 2007; O’Dea et al., 2014; Zhuravlev & Wood, 2008). The aim of these studies is to understand how marine ecosystems and organisms react to different climate-related stressors (Dupont & Pörtner, 2013).

The increase of atmospheric CO_2_ does not only cause elevated dissolved CO_2_ concentration and decrease in sea surface pH, but also a decrease in saturation with respect to calcium carbonate. Since the onset of the industrial revolution (Talmage & Gobler, 2010), the sea surface pH has decreased by 0.1 units (Caldeira & Wickett, 2003) and it is likely to decline another 0.6 units by 2100 and a reduction of carbonate ion-concentration of surface seawater by nearly 50% (Ilyina et al., 2010).

An experimental study suggests that the shell of holoplanktic gastropods dissolves rapidly when surface water becomes undersaturated with respect to aragonite (Feely et al., 2004). As demonstrated by Tajika et al. (2018) juvenile ammonoids are ecologically comparable to holoplanktic gastropods. Recent studies document that some marine species including coleoids such as *Sepia* are more resistant to rising acidity than others (e.g., Gutowska et al., 2010; Ries et al., 2009). Studies about corals (Veron, 2008), foraminifers (Guinotte & Fabry, 2008), calcified nannoplankton (Erba et al., 2010) and echinoderms (Wood et al., 2008) show a severe impact of ocean acidification on many marine calcifiers. These insights have led some authors to conclude that ocean acidification may result in metabolic depression followed by reduced growth (Michaelidis et al., 2005), reduced calcification rates (Hautmann, 2006), hypermetabolism (Michaelidis et al., 2007), or a simultaneous increase of calcification rate and metabolic rates (Wood et al., 2008). Ries et al. (2009) described that marine calcifiers will not only have to face inhibition of calcification due to a reduction of CO_3_^2−^, but also the danger of dissolution of their shell or exoskeleton. Similarly, Bednaršek et al. (2020) observed that the exoskeleton of calcified areas surrounding the neuritic canals of mechanoreceptor in Dungeness crabs was dissolved in present-day ocean acidification in situ conditions. In turn, if there is a correlation between evolutionary changes in septal thickness and phases of ocean acidification, this would confirm that ocean acidification causes evolutionarily relevant ecological stress.

Compared to the number of studies focusing on modern organisms in response to ocean acidification, there is much less knowledge about past impacts of ocean acidification on extinct organisms. O’Dea et al. (2014) examined fossil coccolithophorids, which showed declining calcification rates in populations of *Toweius pertusus* and *Coccolithus pelagicus.* This indicates a species-specific adaptive response to environmental change during the Palaeocene–Eocene Thermal Maximum. In addition, they detected a thinning of *C. pelagicus* coccolithophorids possibly caused by ocean acidification. Arkhipkin and Laptikhovsky (2012) hypothesise that ocean acidification affected planktonic hatchlings and early juveniles of ammonites and belemnites and also impacted fertilisation and growth during short-term ocean acidification events in the Mesozoic.

Past ocean acidification could have been an important or even dominant cause of some marine mass extinctions such as that at the end of the Triassic (Hautmann, 2004; Hautmann et al., 2008; Martindale et al., 2012). There is an increasing consensus that this event was caused by the volcanic activity of the Central Atlantic Magmatic Province (Pálfy, 2003). Estimates of maximum *p*CO_2_ during the end-Triassic mass extinction vary between 2750 ppmv, based on measurements of stomatal densities of land plants (Bonis et al., 2010), and 4400 ppmv, based on stable isotopic values of pedogenic carbonates (Schaller et al., 2011). For comparison, present-day seawater would become undersaturated with respect to aragonite at CO_2_ concentrations between 1200 and 1700 ppmv and additionally with respect to calcite between 1900 and 2800 ppmv (Feely et al., 2004).

Here, we examine the effect of environmental changes around the Triassic–Jurassic boundary on ammonoids. Temporal and evolutionary changes in the ontogenetic change of septal thickness were compared to changes of environmental factors, which potentially influenced the thickness of ammonoid septa. Accordingly, we tested (1) whether elevated CO_2_- and decreasing pH-levels correlate with a decrease in ammonite septal thickness development (tested for the Triassic–Jurassic boundary); (2) whether ocean acidification has a higher effect in colder water due to the enhanced solubility of CO_2_, i.e., we examined the relation between septal thickness and palaeolatitudinal occurrences; (3) whether ontogenetic septal thickness-trajectories reflects palaeogeographic origin, and (4) phylogenetic relationships.

## Material and methods

### Morphometry

**Fig. 1:**
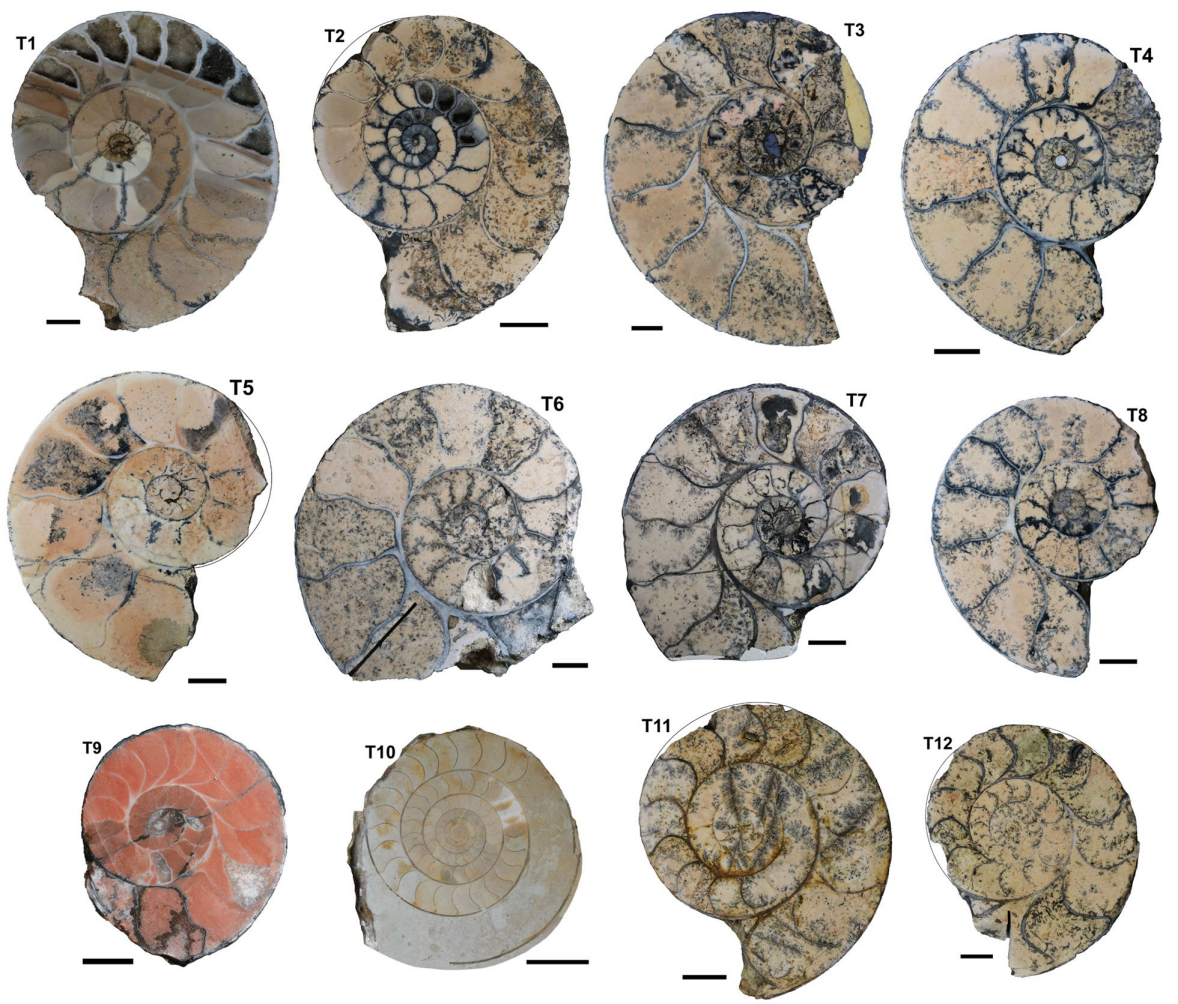
Triassic ammonoids (for details see Table 1). (T1) *Monophyllites* sp. (PIMUZ 37660), (T2) *Halorites* sp. (PIMUZ H.s.T 20), (T3) *Rhacophyllites neojurensis* (PIMUZ 37661), (T4–T8) *Discophyllites ebneri*, (T5) (PIMUZ 37658), (T6) (PIMUZ R.n. 34), (T7) (PIMUZ R.n. 29), (T8) (PIMUZ R.n. 21), (T9) *Megaphyllites* sp., (PIMUZ 37659), (T10) *Arcestes* sp. (PIMUZ 019104 X10 RA4a), (T11) *Cladiscites* sp. (PIMUZ 37662), (T12) *Cladiscites* sp. (PIMUZ 37663). Scale bar = 1 cm

**Fig. 2:**
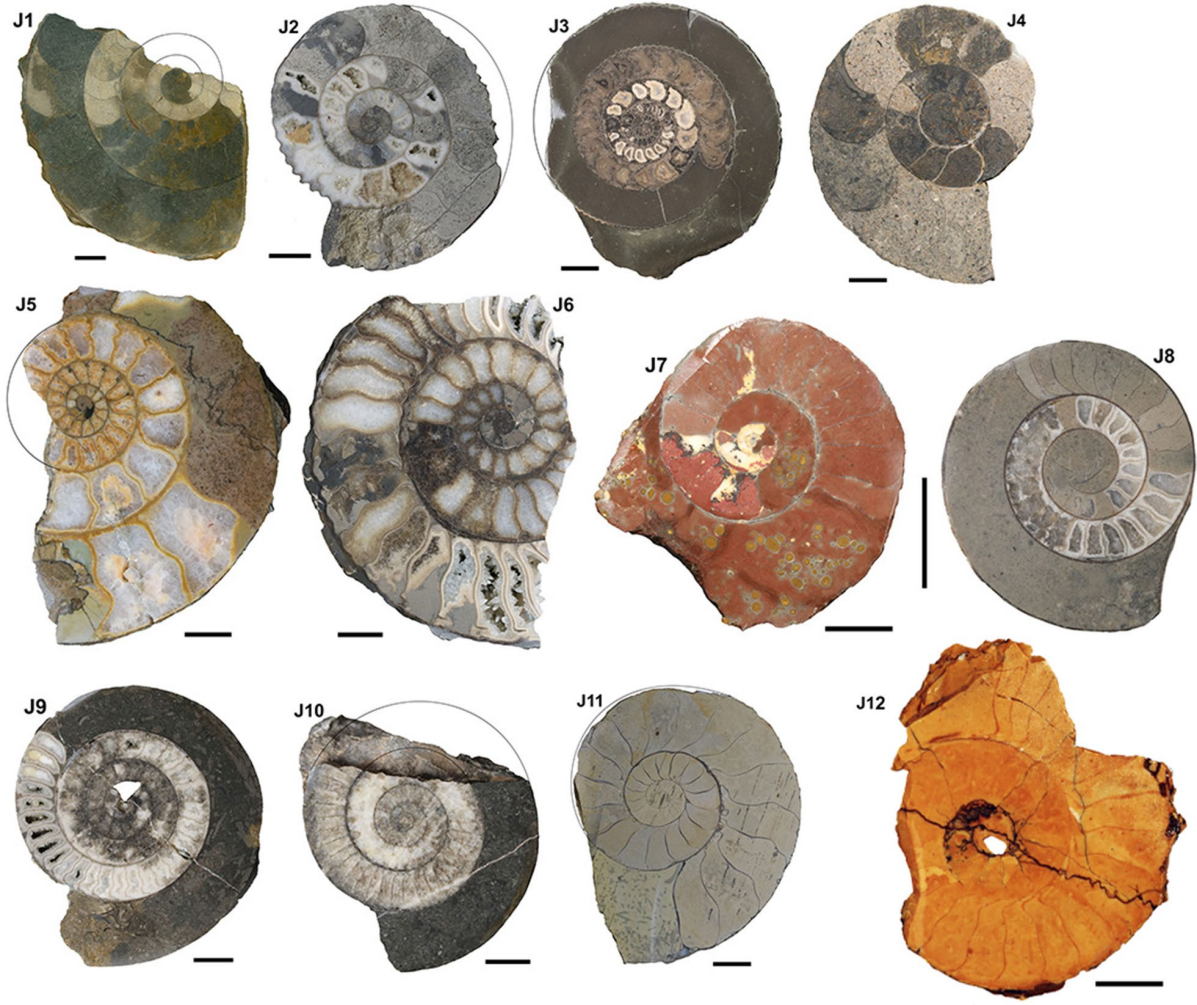
Jurassic ammonoids, Hettangian to Aalenian (for details see Table 1). (J1, J2) *Arietites* sp. (J1) PIMUZ 6734, (J2) PIMUZ 37668, (J3) *Dactylioceras commune* (PIMUZ 13653; 0827), (J4) *Asteroceras* sp. (PIMUZ 13,007), (J5), *Psiloceras naumanni* (PIMUZ 12596), (J6) *Schlotheimiia* sp. (PIMUZ 12,618), (J7) *Leioceras* sp. (PIMUZ 37666), (J8–J10) *Psiloceras planorbis* (PIMUZ 12597; L/1207), (J9) PIMUZ 12597; L/1206), (J10) PIMUZ 12600, (J11), *Lytoceras fimbriatum* (PIMUZ 37665), (J12) *Fuciniceras* cf. *isseli* (PIMUZ 37733). Scale bar = 1 cm

**Fig. 3:**
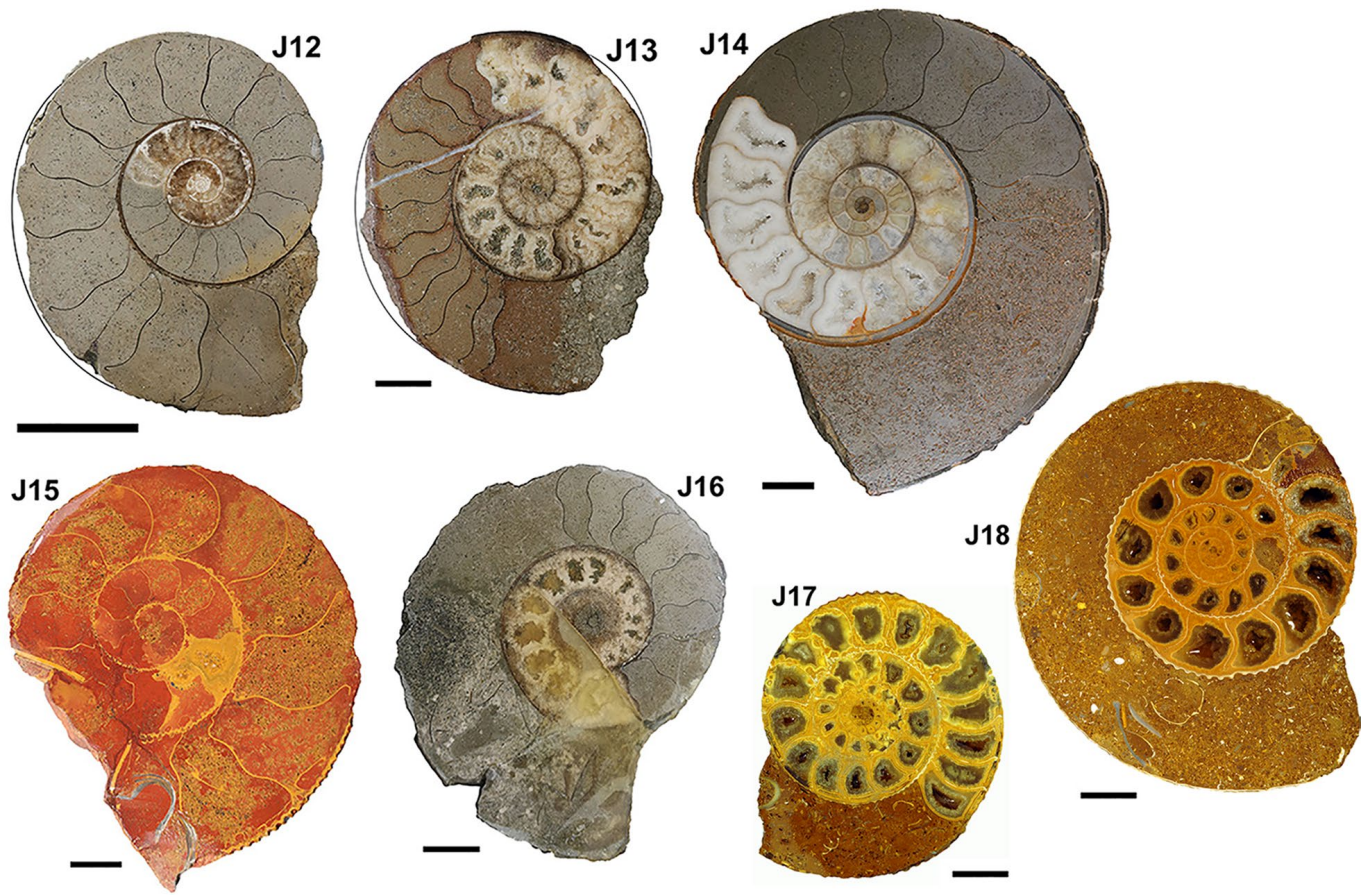
Jurassic ammonoids, Aalenian to Oxfordian (for details see Table 1). (J12) *Ludwigia bradfordensis* (PIMUZ 19067), (J13) *Staufenia opalinoides* (PIMUZ 19087), (J14) *Lud. bradfordensis* (PIMUZ 2034), (J15) *Macrocephalites compressus* (PIMUZ 37667), (J16) *Staufenia opalinoides* (PIMUZ 19091; L/1026), (J17, J18) *Divisosphinctes besairei* (PIMUZ 37734, 37735). Scale bar = 1 cm (except J13: scale bar = 2 cm)

**Fig. 4:**
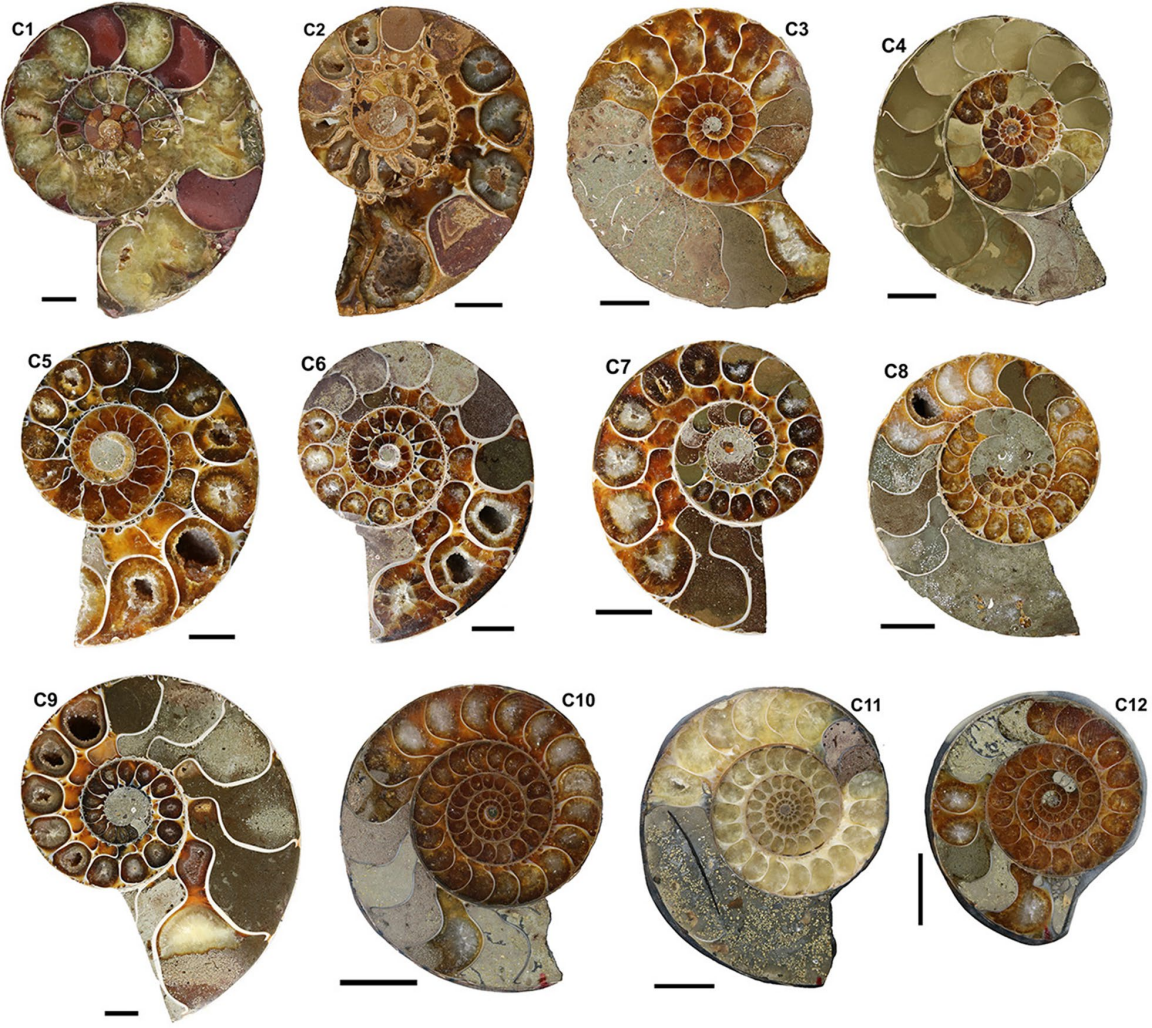
Cretaceous ammonoids, Albian, Madagascar (for details see Table 1). (C1, C2, C5, C7, C9) *Argonauticeras besairei*. (C1), PIMUZ 31258, (C2) (PIMUZ 37664), (C3) *Cleoniceras* sp. RUB-Pal 14106-2G, (C4) *Desmoceras* sp. (RUB-Pal 14107-2H), (C5) RUB-Pal 14102, (C6) *Gaudryceras* sp. (RUB-Pal), (C7) RUB-Pal 14103, (C8 to C12) *Eogaudryceras umbilicostriatus*, (C8) RUB-Pal 14101C, (C9) RUB-Pal 14104–3.11b, (C10) RUB-Pal 14101A, (C11) RUB-Pal 14101B, (C12) RUB-Pal 14101D. Scale bar = 1 cm

**Fig. 5:**
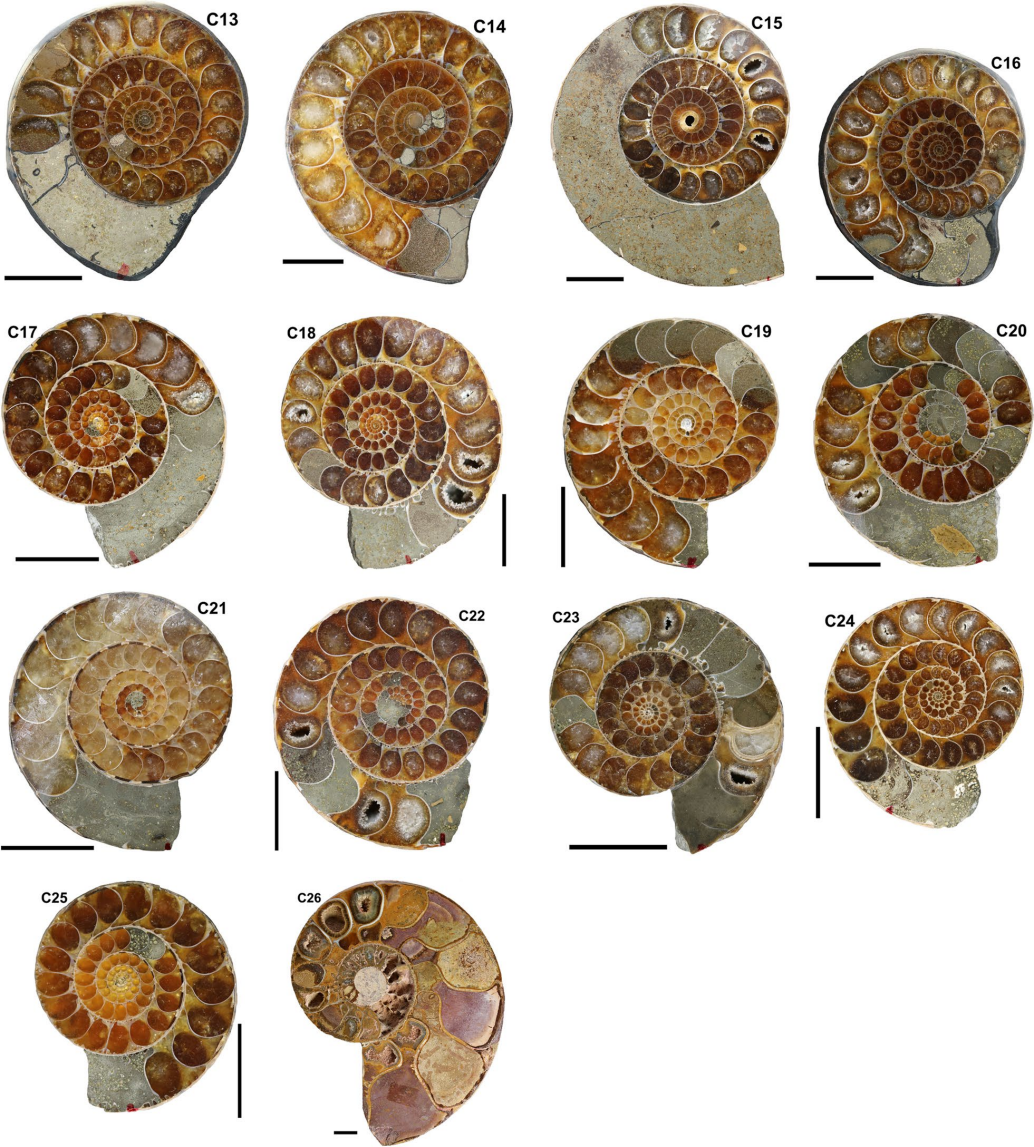
Cretaceous ammonoids, Albian, Madagascar (for details see Table 1). (C13 to C25) *Eogaudryceras umbilicostriatus* (RUB-Pal 14101D), (C13) RUB-Pal 14101E, (C14) RUB-Pal 14101F, (C15) RUB-Pal 14101H, (C16) RUB-Pal 14101G, (C17) RUB-Pal 14101J, (C18) RUB-Pal 14101K, (C19) RUB-Pal 14101M, (C20) RUB-Pal 14101L, (C21) RUB-Pal 14101P, (C22) RUB-Pal 14101Q, (C23) RUB-Pal 14101R, (C24) RUB-Pal 14101T, (C25) RUB-Pal 14101U, (C26) *Argonauticeras besairei* (RUB-Pal 14105). Scale bar = 1 cm

Out of hundreds of Mesozoic specimens, about 100 ammonoids were selected, out of which 57 show a sufficient preservation, where the septal thickness of more than five septa could be measured across the entire whorl cross-section in the plain of symmetry (Table 1). Morphometric measurements from these 57 representatives of various Mesozoic ammonoid families were taken. Thirty-three of these specimens are deposited in the Palaeontological Institute and Museum of Zurich (Switzerland; PIMUZ numbers; Figs. 1, 2, 3, 4). The other 24 specimens are stored in the Institute of Geology, Mineralogy and Geophysics at the Ruhr-Universität Bochum (Germany; RUB-Pal numbers; Figs. 4, 5). All specimens included in this study are well-preserved, showing septa through much of their ontogeny. Slightly incomplete specimens were partially reconstructed by Adobe Illustrator (Adobe Inc.) to measure the diameter. Obliquely cut specimens and poorly preserved specimens (e.g., without or with only a few septa) were excluded.

**Table 1:** Catalogue of all specimens used in this study

Nr	ID. Nr	Species	Family	Period	Stratigraphy	Origin
1	PIMUZ R.n.21	*Discophyllites ebneri*	Phylloceratidae	Triassic (Norian)	Unknown	Timor
2	PIMUZ R.n.22	*Discophyllites ebneri*	Phylloceratidae	Triassic (Norian)	Unknown	Timor
3	PIMUZ R.n.29	*Discophyllites ebneri*	Phylloceratidae	Triassic (Norian)	Unknown	Timor
4	PIMUZ R.n.34	*Discophyllites ebneri*	Phylloceratidae	Triassic (Norian)	Unknown	Timor
5	PIMUZ 37658	*Discophyllites ebneri*	Phylloceratidae	Triassic (Norian)	Unknown	Timor
6	PIMUZ 37659	*Megaphyllites sp.*	Phylloceratidae	Triassic (Norian)	Unknown	Greece
7	PIMUZ 37660	*Monophyllites sp.*	Phylloceratidae	Triassic (Norian)	Unknown	Timor
8	PIMUZ 37661	*Rhacophyllites neojurensis*	Phylloceratidae	Triassic (Norian)	Unknown	Timor
9	PIMUZ H.s.T 20	*Halorites* sp.	Cladiscitidae	Triassic (Norian)	Unknown	Timor
10	PIMUZ 37662	*Cladiscites* sp.	Cladiscitidae	Triassic (Norian)	Unknown	Timor
11	PIMUZ 37663	*Cladiscites* sp.	Cladiscitidae	Triassic (Norian)	Unknown	Timor
12	PIMUZ 019104 RA 4a X10	*Arcestes* sp.	Arcestidae	Triassic (Norian)	Hallstätter Kalk Fm	Austria (Goisern)
13	PIMUZ 012600	*Psiloceras planorbis*	Psiloceratidae	Early Jurassic (Hettangian)	Psilonotenton-Fm	Germany (Bebenhausen 2)
14	PIMUZ 012597 L/1206	*Psiloceras planorbis*	Psiloceratidae	Early Jurassic (Hettangian)	Psilonotenton-Fm	Germany (Nellingen, Esslingen, BW)
15	PIMUZ 012597 L/1207	*Psiloceras planorbis*	Psiloceratidae	Early Jurassic (Hettangian)	Psilonotenton-Fm	Germany (Nellingen, Esslingen, BW)
16	PIMUZ 012596	*Psiloceras naumanni*	Psiloceratidae	Early Jurassic (Hettangian)	Unknown	Austria (Schreinbach am Wolfgangsee)
17	PIMUZ 012618	*Schlotheimia* sp.	Schlotheimiidae	Early Jurassic (Hettang-Sinemurian)	Bamberg-Fm	Germany (Nürnberg)
18	PIMUZ 013007	*Asteroceras* sp.	Asteroceratidae	Early Jurassic (Sinemurian)	Obtusus-Zone	Germany/France?
19	PIMUZ 37668	*Arietites* sp.	Arietitidae	Early Jurassic (Sinemurian)	Arietenkalk Fm	Switzerland (Klettgau)
20	PIMUZ 006734	*Arietites* sp.	Arietitidae	Early Jurassic (Sinemurian)	Arietenkalk Fm	Switzerland (Frick, Aargau)
21	PIMUZ 37733	*Fuciniceras cf. isseli*	Hildoceratidae	Early Jurassic (Sinemur.)	Unknown	Switzerland (Tessin, Arzo)
22	PIMUZ 37665	*Lytoceras fimbriatum*	Lytoceratidae	Early Jurassic	Unknown	Germany (Schömberg)
23	PIMUZ 013653 0827	*Dactylioceras commune*	Dactylioceratidae	Early Jurassic (Toarcian)	Alum Shale series	Great Britain (Yorkshire)
24	PIMUZ 37666	*Leioceras* sp.	Graphoceratidae	Early/Middle Jurassic (Toarcian-Aalenian)	Unknown	France (Belmont d'Azergues)
25	PIMUZ 002034	*Ludwigia bradfordensis*	Graphoceratidae	Early/Middle Jurassic (Toarcian-Aalenian)	Murchisonae-Zone	Switzerland (Passwang SO)
26	PIMUZ 019067	*Ludwigia bradfordensis*	Graphoceratidae	Middle Jurassic (Aalenian)	Staufensis-Zone	Germany (BW)
27	PIMUZ 019087 L/1024	*Staufenia opalinoides*	Graphoceratidae	Middle Jurassic (Aalenian)	Bradfordensis-Zone	Germany (BW)
28	PIMUZ 019091 6. L/1026	*Staufenia opalinoides*	Graphoceratidae	Middle Jurassic (Aalenian)	Bradfordensis-Zone	Germany (BW)
29	PIMUZ 37667	*Macrocephalites compressus*	Macrocephalitidae	Middle Jurassic (Callovian)	Ifental-Fm	Switzerland (Anwil AG)
30	PIMUZ 37734	*Divisosphinctes besairei*	Perisphinctidae	Late Jurassic (Oxfordian)	Unknown	Madagascar (Sakaraha)
31	PIMUZ 37735	*Divisosphinctes besairei*	Perisphinctidae	Late Jurassic (Oxfordian)	Unknown	Madagascar (Sakaraha)
32	RUB-Pal 14101A	*Eogaudryceras umbilicostriatus*	Lytoceratidae	Cretaceous (early Albian)	Ambarimaninga Fm	Madagascar (Majunga)
33	RUB-Pal 14101B	*Eogaudryceras umbilicostriatus*	Lytoceratidae	Cretaceous (early Albian)	Ambarimaninga Fm	Madagascar (Majunga)
34	RUB-Pal 14101D	*Eogaudryceras umbilicostriatus*	Lytoceratidae	Cretaceous (early Albian)	Ambarimaninga Fm	Madagascar (Majunga)
35	RUB-Pal 14101E	*Eogaudryceras umbilicostriatus*	Lytoceratidae	Cretaceous (early Albian)	Ambarimaninga Fm	Madagascar (Majunga)
36	RUB-Pal 14101F	*Eogaudryceras umbilicostriatus*	Lytoceratidae	Cretaceous (early Albian)	Ambarimaninga Fm	Madagascar (Majunga)
37	RUB-Pal 14101G	*Eogaudryceras umbilicostriatus*	Lytoceratidae	Cretaceous (early Albian)	Ambarimaninga Fm	Madagascar (Majunga)
38	RUB-Pal 14101H	*Eogaudryceras umbilicostriatus*	Lytoceratidae	Cretaceous (early Albian)	Ambarimaninga Fm	Madagascar (Majunga)
39	RUB-Pal 14101J	*Eogaudryceras umbilicostriatus*	Lytoceratidae	Cretaceous (early Albian)	Ambarimaninga Fm	Madagascar (Majunga)
40	RUB-Pal 14101K	*Eogaudryceras umbilicostriatus*	Lytoceratidae	Cretaceous (early Albian)	Ambarimaninga Fm	Madagascar (Majunga)
41	RUB-Pal 14101L	*Eogaudryceras umbilicostriatus*	Lytoceratidae	Cretaceous (early Albian)	Ambarimaninga Fm	Madagascar (Majunga)
42	RUB-Pal 14101M	*Eogaudryceras umbilicostriatus*	Lytoceratidae	Cretaceous (early Albian)	Ambarimaninga Fm	Madagascar (Majunga)
43	RUB-Pal 14101P	*Eogaudryceras umbilicostriatus*	Lytoceratidae	Cretaceous (early Albian)	Ambarimaninga Fm	Madagascar (Majunga)
44	RUB-Pal 14101Q	*Eogaudryceras umbilicostriatus*	Lytoceratidae	Cretaceous (early Albian)	Ambarimaninga Fm	Madagascar (Majunga)
45	RUB-Pal 14101R	*Eogaudryceras umbilicostriatus*	Lytoceratidae	Cretaceous (early Albian)	Ambarimaninga Fm	Madagascar (Majunga)
46	RUB-Pal 14101T	*Eogaudryceras umbilicostriatus*	Lytoceratidae	Cretaceous (early Albian)	Ambarimaninga Fm	Madagascar (Majunga)
47	RUB-Pal 14101U	*Eogaudryceras umbilicostriatus*	Lytoceratidae	Cretaceous (early Albian)	Ambarimaninga Fm	Madagascar (Majunga)
48	RUB-Pal?	*Eogaudryceras umbilicostriatus*	Lytoceratidae	Cretaceous (early Albian)	Ambarimaninga Fm	Madagascar (Majunga)
49	RUB-Pal?	*Gaudryceras* sp.	Lytoceratidae	Cretaceous (early Albian)	Ambarimaninga Fm	Madagascar (Majunga)
50	RUB-Pal 14102	*Argonauticeras besairei*	Lytoceratidae	Cretaceous (early Albian)	Ambarimaninga Fm	Madagascar (Majunga)
51	RUB-Pal 14103	*Argonauticeras besairei*	Lytoceratidae	Cretaceous (early Albian)	Ambarimaninga Fm	Madagascar (Majunga)
52	RUB-Pal 14105	*Argonauticeras besairei*	Lytoceratidae	Cretaceous (early Albian)	Ambarimaninga Fm	Madagascar (Majunga)
53	RUB-Pal 14104–3.11b	*Argonauticeras besairei*	Lytoceratidae	Cretaceous (early Albian)	Ambarimaninga Fm	Madagascar (Majunga)
54	PIMUZ 31258	*Argonauticeras besairei*	Lytoceratidae	Cretaceous (early Albian)	Ambarimaninga Fm	Madagascar (Majunga)
55	PIMUZ 37664	*Argonauticeras besairei*	Lytoceratidae	Cretaceous (early Albian)	Ambarimaninga Fm	Madagascar (Majunga)
56	RUB-Pal 14106-2G	*Cleoniceras* sp.	Cleoniceratidae	Cretaceous (early Albian)	Ambarimaninga Fm	Madagascar (Majunga)
57	RUB-Pal 14107-2H	*Desmoceras* sp.	Desmoceratidae	Cretaceous (early Albian)	Ambarimaninga Fm	Madagascar (Majunga)

We also considered measuring the thickness of the outer shell. We refrained from this approach, because the outer shell is usually incompletely preserved since it broke off or it is corroded. Concerning the inner whorls, we found that it is usually impossible or very difficult to differentiate optically between the ventral and dorsal shell (only in nacreous or excellently preserved replacements shells, the dorsal shell can be told apart visually from that of the subsequent whorl). Due to the accordingly high risk of wrong measurements, we decided to exclude measurements of the thickness of the shell-tube.

It is commonly argued that shell thickness may increase during recrystallisation of aragonite to calcite. We assume that shell thickness was not affected because in most cases, the space was simply not available for excess volume to be occupied by the recrystallised shell. Hence, we think that it does not influence our result whether the shell is preserved in pristine aragonite or recrystallised calcite. This is supported by the fact that the highest values in septal slope factors were obtained from specimens with aragonitic shells, although a volume increase happens during the transformation to calcite if space is freely available, which is not the case when the chambers are filled by sediment or cement.

All ammonites were cut with a diamond-bladed saw in the plain of symmetry and polished until the siphuncle was visible in most whorls in order to ensure that they were cut as close to the plain of symmetry as possible. Only septa oriented roughly perpendicularly to the polished surface were measured in order to eliminate biases due to thickness distortion. The ammonite sections were photographed in high resolution. The single septa were measured in the photos in Adobe PhotoShop (version 21.1; Adobe Inc.). Morphometric measurements include data of all visible and intact septa, diameters, and whorl heights.

**Fig. 6:**
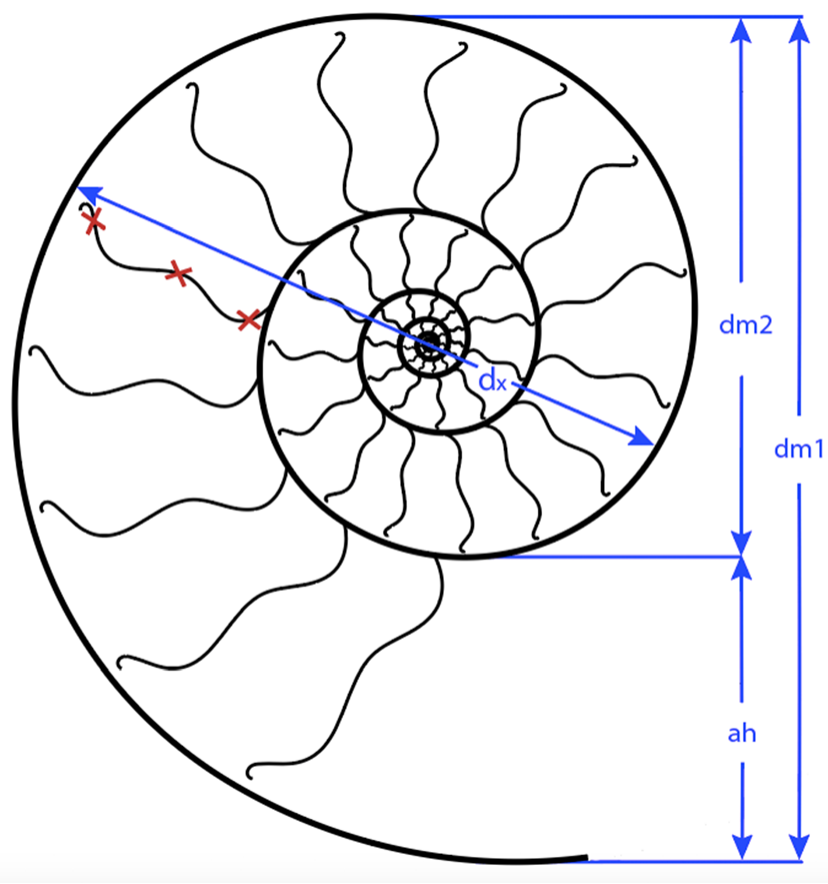
Descriptive terms for conch morphology in longitudinal section. dm1: whole diameter. ah: apertural height, dm_2_ = dm_1_ – ah. dx: Diameter at a specific septum. Red crosses stand representative for the three thickness measurement points at each septum. Descriptive terms after Korn (2010)

**Fig. 7:**
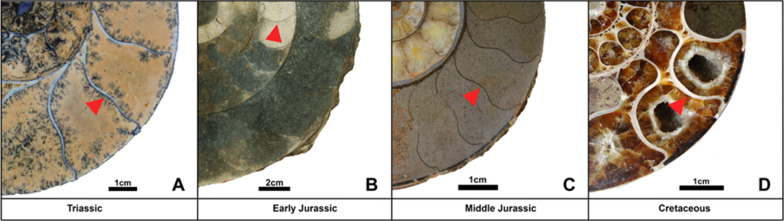
Examples of different septal thickness ranges corresponding to their time period. The figure shows mainly two different septal thicknesses. Ammonoids from the Late Triassic and the Early Cretaceous show a greater septal thickness than those from the Early and Middle Jurassic. **A** *Rhacophyllites neojurensis* (Phylloceratidae); **B** *Arietites* sp. (Arietitidae); **C** *Ludwigia bradfordensis* (Graphoceratidae); **D** *Argonauticeras besairei* (Lytoceratidae). Well discernible examples of septa are indicated by red arrows

Ammonoids show a great variety of septal morphologies. Therefore, septal thicknesses were measured at the inner, middle and outer portion (read crosses in Fig. 6) in order to account for this variability and changes in inclination from dorsal to ventral. The centre of the septum is generally the thickest part. Ammonoids with ammonitic sutures have a documented septal thickness at the margins that is five times lower than at the centre of the same septum (Hewitt & Westermann, 1987) and possibly even more in some species. In addition, the conch diameter was measured at the position of each septum (dx in Figs. 6, 7). For further analyses, the mean thickness was calculated for each septum. The maximum standard deviation between septal measurements is ± 0.0065. The average septal thickness used in this study differs from that used by Westermann (1975). He measured the thickness near the centre of the septum using transverse (perpendicular to the plain of symmetry) rather than sagittal sections. In order to include values from mature conchs, the largest whorls of each specimen were used. Direct measurements of septal thickness are rarely reported in the literature. Most of the studies are restricted to the relationship of septal thickness and sutural complexity^0^ (Saunders, 1995; Westermann, 1975) as well as mode of life (Mutvei, 1975) and neither the impact of changing ocean water chemistry nor systematic position or the palaeogeographic origin (palaeolatitude) was considered.

In total, septa of 57 specimens representing three Triassic families, seven Jurassic families and three Cretaceous families were measured (Table 1). The majority of specimens from the Triassic and Cretaceous come from tropical to subtropical palaeolatitudes, while most Jurassic specimens lived at lower palaeolatitudes (Table 2).

**Table 2:** Species categorised after geographic origin and period with the corresponding septal slope factor (short: septal slope) and palaeolatitude (after Kocsis & Scotese, 2020)

Species	Origin	Latitude	Septal slope	Period
*Arcestes* sp.	Austria (Goisern)	45	0.0018	Late Triassic (Norian)
*Cladiscites* sp.	Timor	− 25	0.0147	Late Triassic (Norian)
*Halorites* sp.	Timor	− 25	0.0098	Late Triassic (Norian)
*Megaphyllites* sp.	Greece	40	0.0054	Late Triassic (Norian)
*Discophyllites ebneri*	Timor	− 25	0.0077	Late Triassic (Norian)
*Monophyllites* sp.	Timor	− 25	0.0067	Late Triassic (Norian)
*Rhacophyllites neojurensis*	Timor	− 25	0.0075	Late Triassic (Norian)
*Psiloceras planorbis*	Germany	50	0.0047	Early Jurassic (Hettangian)
*Psiloceras naumanni*	Austria (Schreinbach am Wolfgangsee)	45	0.0034	Early Jurassic (Hettangian)
*Arietites* sp.	Switzerland	50	0.0012	Early Jurassic (Sinemurian)
*Asteroceras* sp.	Germany/France	50	0.0019	Early Jurassic (Sinemurian)
*Schlotheimia* sp.	Germany (Nürnberg)	50	0.0036	Early Jurassic (Hettangian-Sinemurian)
*Fuciniceras* cf. *isseli*	Switzerland (Tessin, Arzo)	45	0.0064	Early Jurassic (Sinemur.)
*Dactylioceras commune*	Great Britain (Yorkshire)	50	0.0037	Early Jurassic (Toarcian)
*Lytoceras fimbriatum*	Germany (Schömberg b. Balingen)	50	0.0067	Early Jurassic
*Leioceras* sp.	France (Belmont d'Azergues)	50	0.001	Early/Middle Jurassic (Toarcian, Aalenian)
*Ludwigia bradfordensis*	Switzerland/Germany	50	0.0021	Middle Jurassic (Aalenian)
*Staufenia opalinoides*	Germany (Baden-Württemberg)	50	0.0004	Middle Jurassic (Aalenian)
*Macrocephalites compressus*	Switzerland (Anwil, Aargau)	45	0.0041	Middle Jurassic (Callovian)
*Divisosphinctes besairei*	Madagascar (Sakaraha)	− 20	0.0037	Late Jurassic (Oxfordian)
*Argonauticeras besairei*	Madagascar (Majunga)	− 40	0.0107	Early Cretaceous (Albian)
*Cleoniceras* sp.	Madagascar (Majunga)	− 40	0.0028	Early Cretaceous (Albian)
*Desmoceras* sp.	Madagascar (Majunga)	− 40	0.0078	Early Cretaceous (Albian)
*Gaudryceras* sp.	Madagascar (Majunga)	− 40	0.0124	Early Cretaceous (Albian)
*Eogaudryceras umbilicostriatus*	Madagascar (Majunga)	− 40	0.004	Early Cretaceous (Albian)

Errors in our measurements may have different origins. For example, if the specimen is cut slightly obliquely or not in the plain of symmetry, this causes deviations from the accurate values. Also, septal thickness is, in most cases, so low that these measurements already have a certain error. To account for the latter, we took three measurements and took the average value. Furthermore, by using the septal slope factor instead of single values, simple measurement errors are levelled out to some degree.

With the ratio between diameter and septal thickness, a factor was introduced by performing a linear regression to calculate the septal slope factor and its intercept. After calculating this factor for each species and family, this was summarised for each time bin. The resulting septal slope factors further served to visualise differences between these ammonoid taxa. In addition, the septal slope factors were used for statistical analyses to investigate the impact of environmental, geographical and phylogenetic relationships.

Most statistical analyses were carried out in the freely available statistical environment Rstudio. The “ggpubr” R package was used for ggplot2-based data visualisation. For the Spearman correlation rank test, the package “corrplot” was used. Intercept values are not further discussed in this study since X (here values of septal thickness) can never be equal 0.

### Measurement and analysis of environmental parameters

Quantitative values of various factors were obtained from the literature. The assessed factors are CO_2_-content of the atmosphere (Miller et al., 2005; Berner, 2006; Witkowski et al., 2018), sea surface pH-values (Ridgwell, 2005), seawater Mg/Ca ratios and [Ca^2+^] (Arvidson et al., 2011; Demicco et al., 2005; Lowenstein et al., 2014), as well as sea surface temperatures (Grossman & Joachimski, 2020; Miller et al., 2005). Trends of these abiotic factors through time were statistically tested against the age midpoint and the slope value. By performing a non-parametric test for monotonic trend detection, known as the Mann–Kendall test (Helsel & Frans, 2006), we tested for consistently increasing or decreasing trends to compare general trends in data of the abiotic factors.

Time series often show some form of overall trend (rising/ falling) suggesting a possibly wrong positive or negative relationship; it is challenging to test for tendencies of non-parametric and non-monotonic trends (Sang et al., 2018). Thus, before performing any further analyses, it was important to test whether the data follow the bivariate normal distribution sufficiently. For this study, Spearman’s Rank correlation coefficient was used (Spearman, 1904).

Before performing Spearman’s rank correlation test, we examined whether the data are normally distributed (Table 3). For all abiotic factors, the Shapiro–Wilk test for normality was performed (Table 3). Most *p*-values are lower than the predetermined significance level of 0.05, thus implying that the distribution of the data is significantly different from a normal distribution. In other words, we cannot assume the normality of the data (Table 3). The value of *W* lies between zero and one. Since the data of this study are not normally distributed, the Fligner–Killeen test was applied to test for homogeneity of variances. Fligner–Killeen test uses an asymptotic Chi-squared-distributed test-statistic; it is very robust against departures from normalities (Fligner & Killeen, 1976).

**Table 3:** Matrices with results of tests for normality of the data produced using PAST (A, top) and including R-output of the Fligner–Killeen test (B, bottom) of homogeneity of variances (slope is short for septal slope factor)

(A)	SlopeFactor	CO_2_	Latitude_SN	pH
N	25	25	25	25
Shapiro–Wilk *W*	0.9377	0.8945	0.803	0.8347
*p*(normal)	0.1308	0.01399	0.000255	0.000917
Jarque–Bera JB	3.05	0.5951	3.32	2.821
*p*(normal)	0.2176	0.7426	0.1901	0.244
*p*(Monte Carlo)	0.0815	0.672	0.0676	0.0853
Chi^2^	1.4	2.36	2.04	3.96
*p* (normal)	0.23672	0.12448	0.15321	0.046594
Chi^2^ OK (*N* > 20)	Yes	Yes	Yes	Yes
Anderson–Darling A	0.5021	0.9992	1.957	1.519
p(normal)	0.1874	0.01023	3.80E−05	0.000489

(B) Fligner–Killeen test	Chi^2^	Critical value (Chi^2^ table)	Degrees of freedom	*p*-value
Slope ~ age (Ma)	19.00	19.68	11	0.06
Slope ~ sea surface pH	8.67	10.22	8	0.37
Slope ~ [Ca^2+^]	15.74	16.92	9	0.07
Slope ~ Mg/Ca ratio	14.61	14.68	9	0.10
Slope ~ atmospheric CO_2_	17.15	18.31	10	0.07
Age (Ma) ~ Atmospheric CO_2_	3.60	3.94	10	0.96
Age (Ma) ~ sea surface pH	4.19	3.49	8	0.84
Age (Ma) ~ Mg/Ca ratio	4.73	4.17	9	0.86
Age (Ma) ~ [Ca^2+^]	2.80	3.33	9	0.97

The null hypothesis (H_0_) is that the variances in each of the groups are the same. By calculating the p-value, Chi-square and the degrees of freedom, the critical value from the Chi-squared distribution table was determined. The results (Table 3) show that most of the Chi-squared values are smaller than the critical values, i.e., the H_0_ cannot be rejected and heteroscedasticity is assumed.

Often, palaeontological data can only be expressed as time series but in this study, we wanted to test whether two series co-vary or correlate. However, it is not recommended to just use time series because of the risk of false positives (type I errors; McKinney, 1990; see also G. T. Lloyd: http://www.graemetlloyd.com/methgd.html). Therefore, we performed the Generalized differencing of time series by Graeme T. Lloyd (http://www.graemetlloyd.com/methgd.html) to analyse the differences within the values and not between the values by differencing (using the new bin-to-bin as a new time series).

### Measurement and analysis of geographical parameters

To test whether the distribution of included species show a palaeogeographical pattern, we classified them into categories of high and low palaeolatitude occurrences depending on their origin (Tables 2, 3). With the maps of Scotese (2001) and Kocsis and Scotese (2021), palaeolatitudes were estimated and assigned to each species depending on their locality and age. The latitude was plotted against the septal slope factor to test if there is a correlation between the habitat position and the relative septal thickness. The data again were tested for normality (Table 3). Subsequently, we performed the generalised differencing of time series with the Spearman correlation coefficient.

### Measurement and analysis of the systematic context

**Fig. 8:**
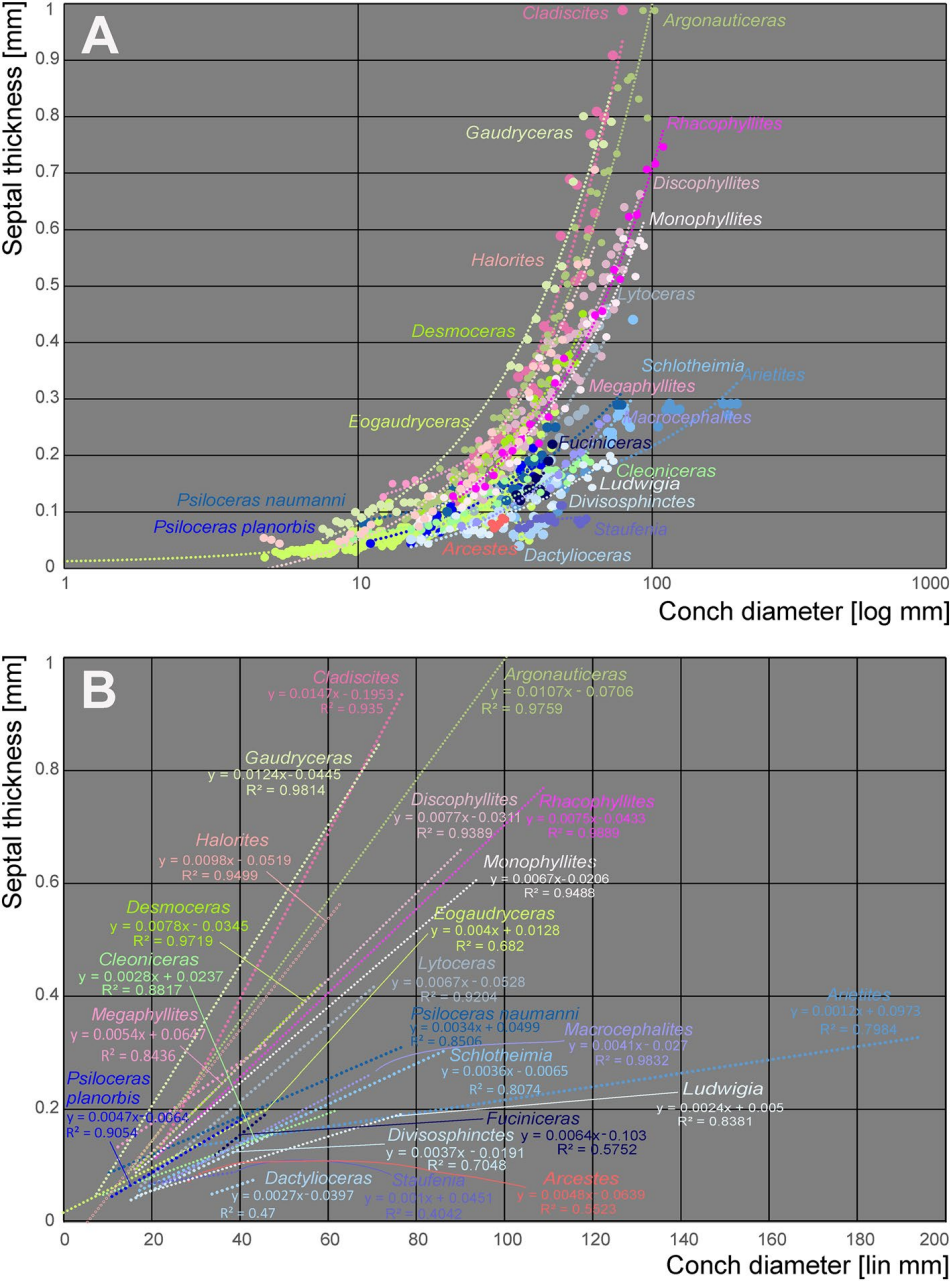
**A** Semilogarithmic biplot of septal thickness versus conch diameter at the measured septa position for all ammonoid genera included. The dashed lines indicate the linear regression lines for each ammonoid family, which are represented by specific colours. **B** Linear biplot of the same data, but only displaying the linear regression lines with their equation and *R*^2^

To test if the relative septal thickness follows a pattern linked with systematic position (thus indirectly reflecting phylogenetic position), the conch diameter and septal thickness were plotted against each other to see if the ratios of species from the same family show closer similarities than between families (Fig. 8). With a linear regression, the septal slope factor and the intercept of each family were calculated and trend lines were added to visualise septal slope factor and intercept.

Analogously, the fossil data were categorised into time groups corresponding to their geologic age using the bins Early, Middle and Late for the Jurassic, while the Triassic and Cretaceous were not further subdivided because we included only Late Triassic and Early Cretaceous ammonoids. The Jurassic was subdivided into Early, Middle and Late to evaluate if there is a difference between species depending on the temporal proximity to the ocean acidification event at the Triassic–Jurassic boundary. Due to the low number of specimens, a finer temporal resolution appeared not feasible.

Unfortunately, a comprehensive phylogenetic analysis of Mesozoic ammonoids is not available and we had to use the coarse schemes of Yacobucci (2015) and Moriya (2015). Only small systematic groups or time intervals have been assessed (e.g., Hardy et al., 2012; Hoffmann, 2010; Zacaï et al., 2017) and thus, conclusive interpretations of possible links between phylogeny and septal thickness are not possible yet. However, the differences in septal thickness between certain groups suggest that there is a phylogenetic aspect (see below).

## Results

In order to compare the septal thickness (Figs. 1, 2, 3, 4, 5, 6, 7) among ammonite taxa, we used the septal slope factor and the intercept of the linear regression for each species and family (see Methods, Tables 2 and 4 for species level; Table 5 for family level); this was summarised for each time interval (Table 6). The resulting septal slope factors of the species served to visualise differences among these ammonoid taxa (Fig. 8) with respect to septal thickness. Furthermore, the septal slope factors were used for statistical analyses to investigate the respective roles of environmental, geographical and phylogenetic factors.

**Table 4:** Slope, intercept and *R*^2^-value of the average septal slope factor of 23 species corresponding to their geological age (average value of beginning and end of the corresponding stage after the ICS from 2020, Cohen et al., 2013). For the raw data see Additional file 1

Species	Septal slope factor	Intercept	Age (Ma, midpoint)	*R*^2^ of average septal slope factor
*Desmoceras* sp*.*	0.0078	− 0.0345	105.5	0.9719
*Cleoniceras* sp.	0.0028	− 0.0237	105.5	0.8817
*Gaudryceras* sp.	0.0124	− 0.0445	105.5	0.9814
*Argonauticeras besairei*	0.0107	− 0.0706	105.5	0.9759
*Eogaudryceras umbilicostriatus*	0.004	0.0128	106.75	0.682
*Divisosphinctes besairei*	0.0037	− 0.0191	154.3	0.7048
*Macroceph. compressus*	0.0041	− 0.027	168.8	0.9832
*Ludwigia bradfordensis*	0.0024	0.005	172.2	0.8381
*Staufenia opalinoides*	0.0004	0.0689	172.2	0.0837
*Leioceras* sp.	0.001	0.0451	176.5	0.4042
*Dactylioceras commune*	0.0027	− 0.0397	178.4	0.47
*Lytoceras fimbriatum*	0.0067	− 0.0528	187.7	0.9204
*Asteroceras* sp.	0.0019	0.0019	195.05	0.8359
*Arietites* sp.	0.0012	0.0973	195.05	0.7984
*Fuciniceras* cf. *isseli*	0.0064	− 0.0103	195.05	0.5752
*Schlotheimia* sp.	0.0036	− 0.0065	196.1	0.8074
*Psiloceras planorbis*	0.0047	− 0.0064	200.3	0.9054
*Psiloceras naumanni*	0.0034	0.0499	200.3	0.8506
*Arcestes* sp.	0.0048	− 0.0639	222.8	0.5523
*Halorites* sp.	0.0098	− 0.0519	222.8	0.9499
*Cladiscites* sp*.*	0.0147	− 0.1953	222.8	0.935
*Discophyllites ebneri*	0.0077	− 0.0311	222.8	0.9389
*Megaphyllites* sp.	0.0054	0.0647	222.8	0.8436
*Monophyllites* sp.	0.0067	− 0.0206	222.8	0.9488
*Rhacophyll. neojurensis*	0.0075	− 0.0433	222.8	0.9889

**Table 5:** Septal slope factor (short: slope) and intercept of ammonoid families covered in this study

Family	Slope	Intercept
Arcestidae	0.0048	− 0.0639
Arietitidae	0.0012	0.0973
Asteroceratidae	0.0019	0.0019
Cladiscitidae	0.0123	− 0.1236
Cleoniceratidae	0.0028	− 0.0237
Dactylioceratidae	0.0027	− 0.0397
Desmoceratidae	0.0078	− 0.0345
Graphoceratidae	0.0013	0.0397
Hildoceratidae	0.0064	− 0.0103
Lytoceratidae	0.0085	− 0.0388
Macrocephalitidae	0.0041	− 0.027
Perisphinctidae	0.0037	− 0.0191
Phylloceratidae	0.0068	− 0.0076
Psiloceratidae	0.0041	0.0218
Schlotheimiidae	0.0036	− 0.0065

**Table 6:** Septal slope factor (short: slope) and intercept of the examined time bins

Age	N species	Slope	Intercept
*Late Triassic*	7	0.0081	− 0.0488
*Early Jurassic*	8	0.0038	0.0042
*Middle Jurassic*	4	0.0020	0.023
*Late Jurassic*	1	0.0037	− 0.0191
*Early Cretaceous*	5	0.0075	− 0.0321

### Environmental parameters

Abiotic factors used in this study are listed according to their corresponding time bins including the data sources (Table 7). This selection of factors is not exhaustive, relations to other factors may be examined in the future.

**Table 7:** States of four abiotic factors correlated with age midpoint (average of ages of the beginning and the end of the corresponding stages after international chronostratigraphic chart from stratigraphy.org) and septal slope factor of ontogenetic trajectory of septal thickness through the Mesozoic. Palaeoenvironmental data are from Berner (2006): CO_2_, Demicco et al. (2005): Mg/Ca ratio, [Ca^2+^] (mol mol^−1^), and Ridgwell (2005): Sea surface pH

Species	Age midpoint (Ma) Cohen et al. (2013)	Septal slope factor (this study)	CO_2_ (ppm) Berner et al. (2006)	CO_2_ (ppm) Berner et al. (2006)	Mg/Ca ratio (mol mol^−1^) Demicco et al. (2005)	[Ca^2+^] (mol mol^−1^) Demicco et al. (2005)	Sea surface pH Ridgwell (2005)
*Desmoceras*	105.5	0.0078	1650	1033.87	0.6	5.15	7.55
*Cleoniceras*	105.5	0.0028	1650	1033.87	0.6	5.15	7.55
*Gaudryceras*	105.5	0.0124	1650	1033.87	0.6	5.15	7.55
*Argonauticeras*	105.5	0.0107	1650	1033.87	0.6	5.15	7.55
*Eogaudryceras umbilicostriatus*	106.75	0.004	1700	1033.87	0.6	5.15	7.55
*Divisosphinctes*	154.3	0.0037	2430	693.66	1.45	3.5	7.62
*Macrocephalites compressus*	168.8	0.0041	2430	875.22	1.45	3.5	7.62
*Ludwigia*	172.2	0.0024	2200	963.28	1.51	3.3	7.62
*Staufenia*	172.2	0.0004	2200	963.28	1.51	3.3	7.62
*Leioceras*	176.5	0.001	1950	963.28	1.52	3.25	7.6
*Dactylioceras*	178.4	0.0027	1800	963.28	1.53	3.2	7.57
*Lytoceras*	187.7	0.0067	1650	645.87	1.9	2.9	7.54
*Asteroceras*	195.05	0.0019	1550	870.46	2.4	2.5	7.52
*Arietites*	195.05	0.0012	1550	870.46	2.4	2.5	7.52
*Fuciniceras*	195.05	0.0064	1550	870.46	2.4	2.5	7.52
*Schlotheimia*	196.1	0.0036	1500	870.46	2.45	2.45	7.52
*Psiloceras planorbis*	200.3	0.0047	1100	870.46	2.7	2.3	7.58
*Psiloceras naumanni*	200.3	0.0034	1100	870.46	2.7	2.3	7.58
*Arcestes*	222.8	0.0048	700	1216.61	2.7	2.2	7.7
*Halorites*	226.6	0.0098	750	1216.61	2.6	2.3	7.73
*Cladiscites*	226.6	0.0147	750	1216.61	2.6	2.3	7.73
*Discophyllites*	226.6	0.0077	750	1216.61	2.6	2.3	7.73
*Megaphyllites*	226.6	0.0054	750	1216.61	2.6	2.3	7.73
*Monophyllites*	226.6	0.0067	750	1216.61	2.6	2.3	7.73
*Rhacophyllites neojurensis*	226.6	0.0075	750	1216.61	2.6	2.3	7.73

In this study, we focus on ocean acidification, using *p*CO_2_ (Berner, 2006; Royer, 2014; Witkowski et al., 2018) and sea surface pH estimations (Ridgwell, 2005) from the literature. Additionally, we included seawater Mg/Ca-ratio and [Ca^2+^]-data from Demicco et al. (2005), Arvidson et al. (2011), and Lowenstein et al. (2014) to test for a correlation of septal thickness with times of aragonite and calcite seas. Data of atmospheric CO_2_ were taken from Berner (2006) and Witkowski et al. (2018) and sea surface pH-data from Ridgwell (2005).

**Fig. 9:**
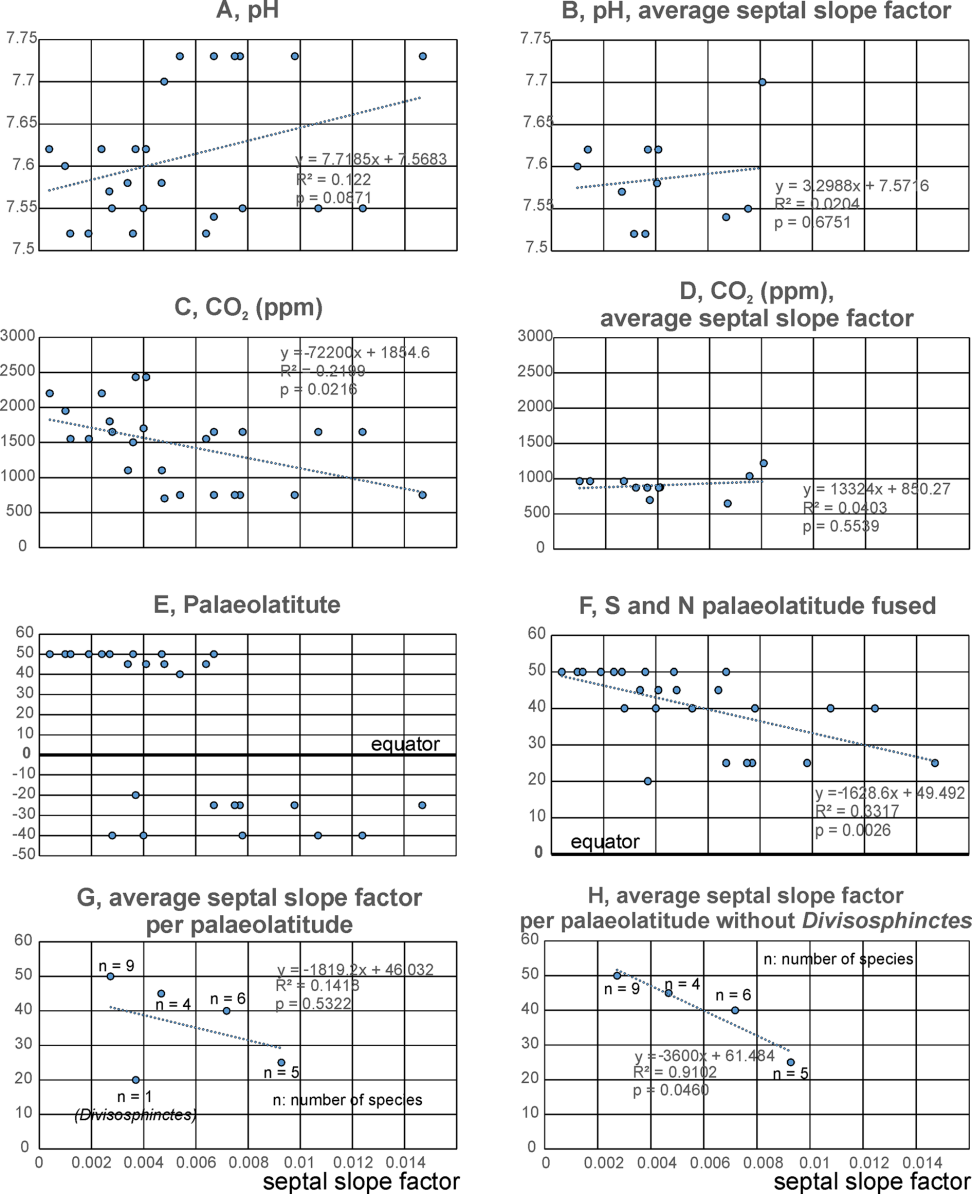
Correlation of abiotic factors (vertical axes) with the septal slope factor (horizontal axis). **A** pH after Ridgwell (2005); **B** like A, but with average septal slope factors. **C** atmospheric CO_2_ (Witkowski et al., 2018). **D** like **C**, but with average septal slope factors. **E** Palaeolatitudal distribution using the maps of Kocsis and Scotese (2021). **F** Like **E**, but northern and southern latitudes fused. **G** Like **F**, but with average septal slope factor per latitude. **H** Like **G**, but without the outlier *Divisosphinctes*

**Fig. 10:**
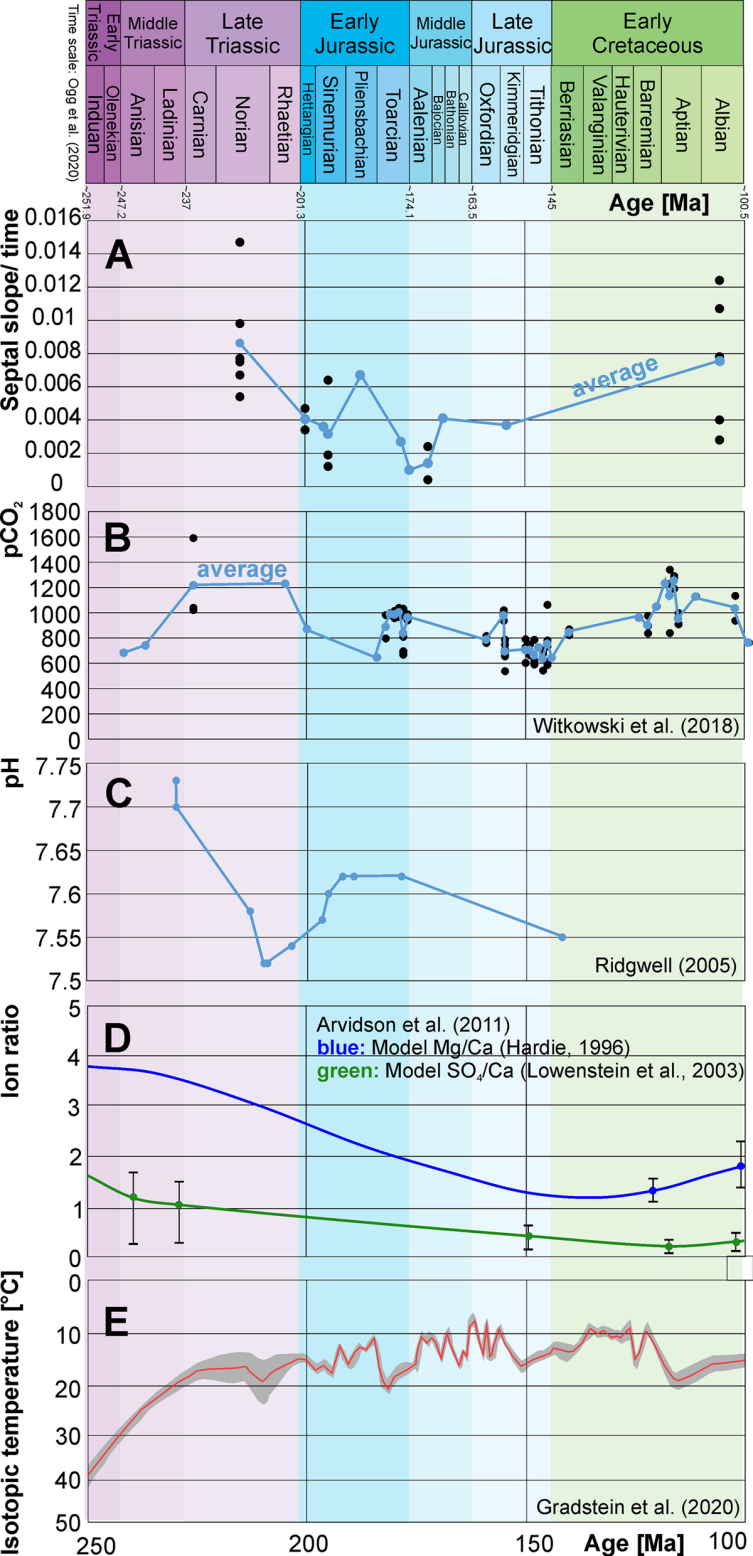
Correlation of abiotic factors, time scale after Cohen et al. (2013). **A** septal slope factor versus time (blue line: the average); **B** atmospheric CO_2_ (Witkowski et al., 2018). **C** isotopic temperature after Veizer and Prokoph (2015. **D** Ion ratios of Mg/Ca and SO_4_/Ca after Arvidson et al. (2011)

There is only poor evidence for a correlation the septal slope factors through ontogeny through the Mesozoic with CO_2_ and pH-levels (Table 8, Figs. 9A–D, 10). In Fig. 10B, the CO_2_-level rises when the septal slope factor increases during the Jurassic and similarly decreases again in the Cretaceous. The Generalized differencing of time model including the Spearman correlation factor of these data revealed no statistically significant correlation (two-sided *p*-value with the values from Witkowski et al., 2018 = 0.506, rho = 0.143; using the values of Berner, 2006, *p*-value = 0.067, rho = − 0.380; Fig. 9C).

**Table 8:** Linear correlation matrix of the septal slope factor and the main abiotic environmental factors

	Septal slope factor	CO_2_	Latitude S/N	pH
SlopeFactor		0.021551	0.00259	0.087074
CO_2_	0.45727		0.008765	1.64E–05
Latitude S/N	− 0.57592	− 0.51278		0.000204
pH	0.34922	0.74909	− 0.67665

From the Late Triassic to the Early Jurassic, the pH decreased with the septal slope factor (Fig. 10C), suggesting a positive correlation of the two factors. While the septal slope factor slightly rose in the Cretaceous (although the number of data points is low), the pH decreased. Nevertheless, the generalised differencing of time series paired with Spearman’s rank correlation test revealed that the *p*-value is 0.066 (two-sided) and rho is very close to 0 (rho = − 0.09), thus indicating no correlation between sea surface pH and septal slope factor. Since the septal slope factor rose sharply in the Early Jurassic in only some groups (the highest dot indicates *Arietites* sp*.*), it is difficult to compare this factor to the abiotic factors. Therefore, it is possible that the significance value is low even when the graph shows that the septal slope factors rose while the pH decreased as expected due to ocean acidification around the Triassic–Jurassic boundary. To sum up, the result is ambiguous regarding the pH and further data are needed.

### Geographical parameters

Specimens from the European Jurassic, which represent the highest northern palaeolatitude in this study, show a tendency towards lower septal slope factors. This means that they have thinner septa relative to their diameter, while specimens from the European and SE-Asian (Timor) Triassic as well as the Cretaceous of Madagascar show higher relative septal thicknesses (Fig. 9E–H).

The data were tested for normality with the Shapiro–Wilk test. The *p*-value of the latitude data lies under the significance-level and the W-value is not close enough to 1 (*W* = 0.80, one-sided *p*-value 0.0003). Consequently, we assume no normality of the data. Subsequently, we performed the Generalized differencing of time series with Spearman’s correlation coefficient (rho = − 0.44, two-sided *p*-value = 0.033) indicating no statistical significance and with a correlation coefficient of rho = − 0.44, a weak degree of association between these two variables is indicated.

### Phylogeny

Septal slope factor and intercept were determined with linear regression models, represented by the lines in different colours in Fig. 8. The results show that each of the sampled families has a specific slope and intercept (Table 5). This septal slope factor was used to estimate the phylogenetic signal of septal thickness.

**Fig. 11:**
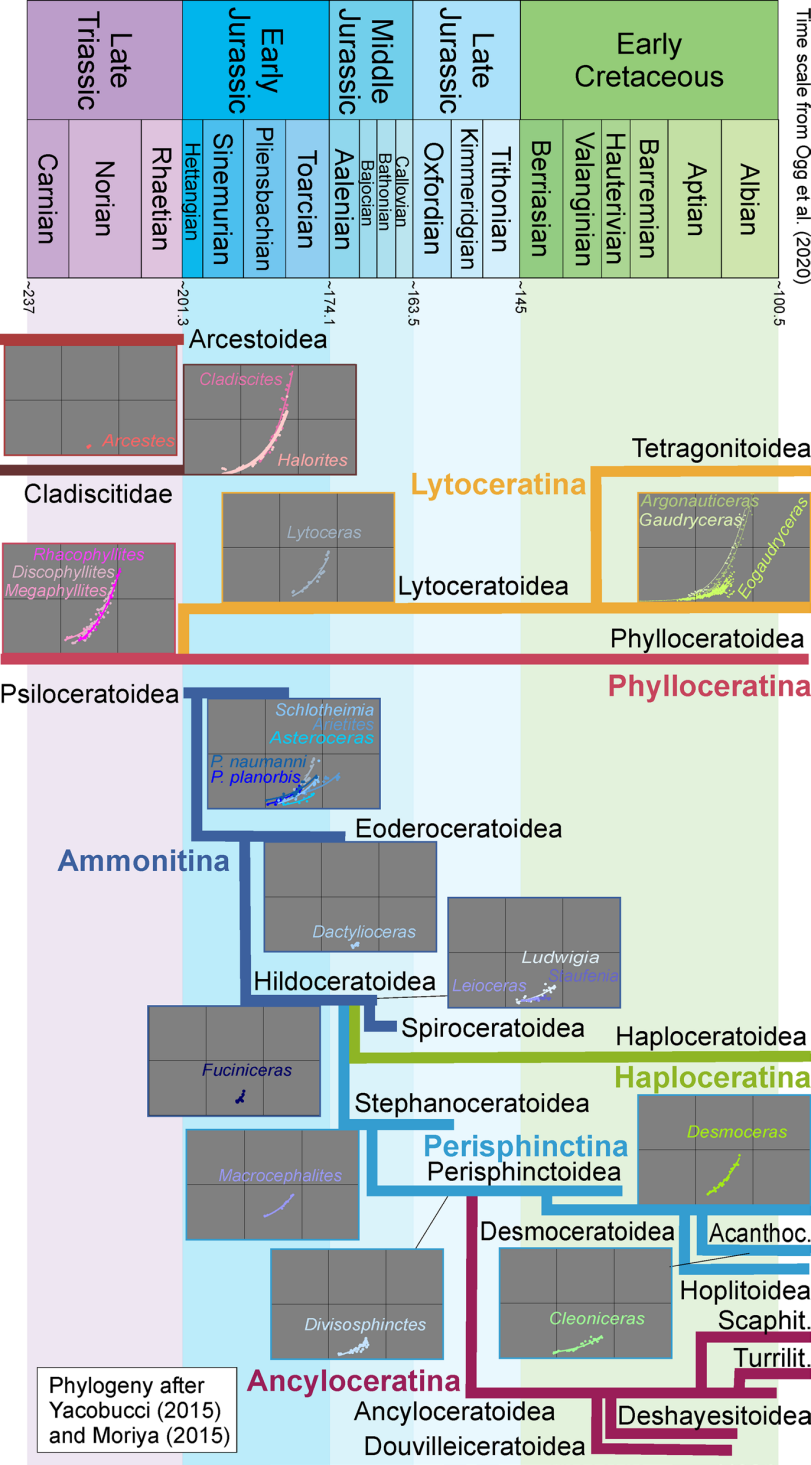
Ranges of Mesozoic ammonoid (super-)families. Possible evolutionary relationships modified after Moriya (2015) and Yacobucci (2015). Septal slope factor diagrams after Fig. 8A

Figure 11 shows a simplified phylogeny of the post-Triassic ammonoids. According to Page (2008), Yacobucci (2015) and Moriya (2015), the Psiloceratina gave rise to all Jurassic and Cretaceous ammonoid lineages except the Phylloceratina and Lytoceratina. The septal slope factors and intercepts of representatives of the included families were calculated by linear regression (Table 5). Cladiscitidae and Phylloceratidae from the Triassic as well as Desmoceratidae and Lytoceratidae from the Cretaceous show the steepest regression line, i.e., thicker septa relative to diameter.

The Early Jurassic *Lytoceras fimbriatum* (Lytoceratidae) has a lower slope factor than its Cretaceous relatives. In addition, taxa of all taxonomic ranks (Ammonitida, Psiloceratidae, *Arietites* sp. and *Lytoceras fimbriatum*) from the Early Jurassic tend to show thinner septa (lower septal slope factors) than those from the Triassic and Cretaceous, but our data encompass only a small selection of orders, families and species. Thus, more data from additional species and further research are required to improve our understanding of the relationships between relative septal thickness and systematic groups.

The same method with the calculation of septal slope factor and intercept with a linear regression model was applied to all sampled taxa for each time bin (Early, Middle and Late Jurassic, Triassic and Cretaceous; Table 6). The steepness of the regression lines indicates the thickness of septa in relation to conch diameter. As visible in Fig. 11, taxa from each time bin have a specific steepness of septal slope and intercept. Early Jurassic taxa revealed the highest septal slope factor, followed by those from the Middle and Late Jurassic as well as the Triassic. Cretaceous ammonites revealed the highest ratios and the flattest slope. A gradual decrease in septal thickness relative to diameter from the Jurassic over the Triassic to the Cretaceous is discernible.

**Fig. 12:**
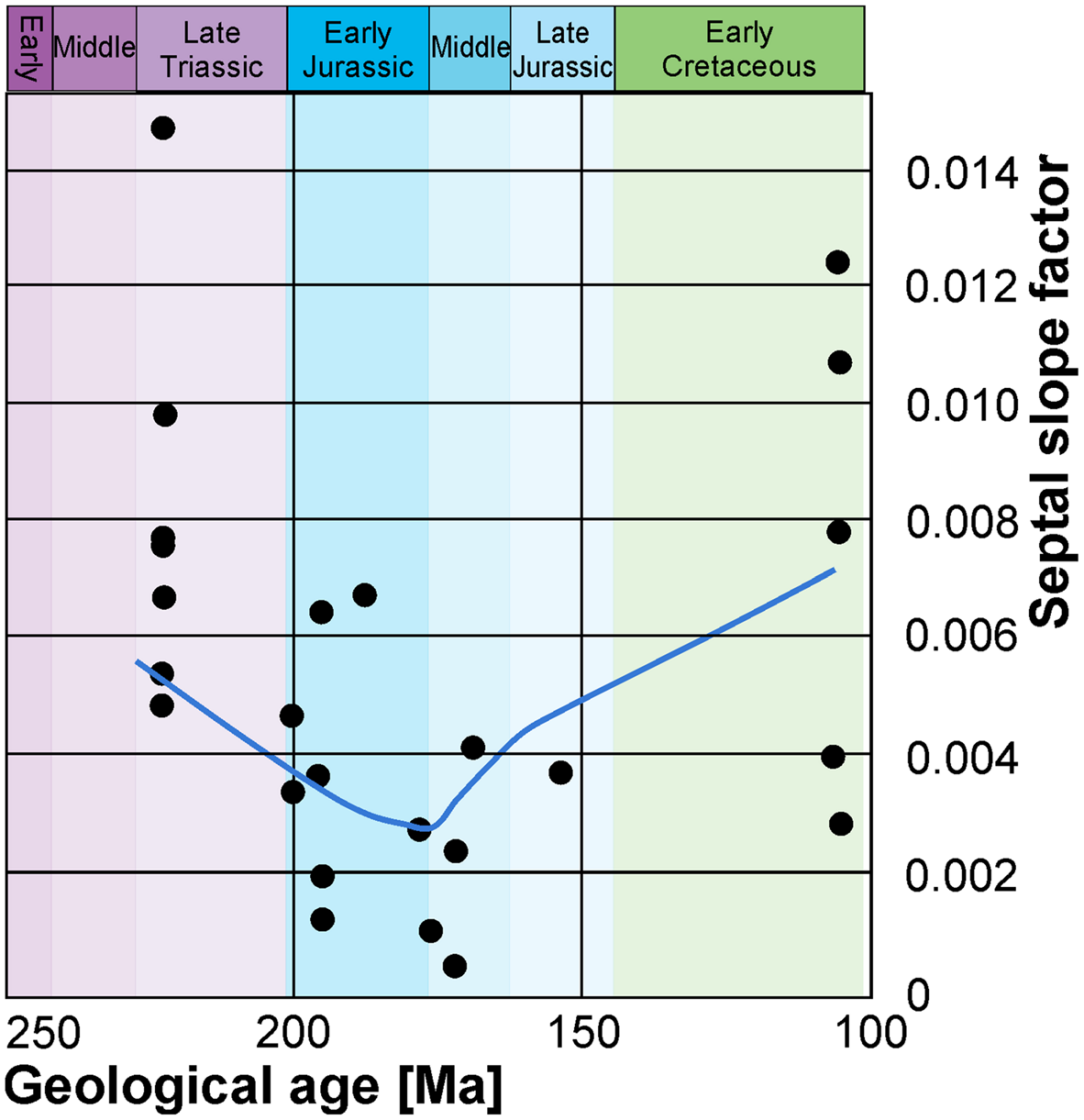
Septal thickness septal slope factor data: Mann–Kendall test, septal slope factor as a function of time. The lowest point is *Arietites* sp. The graph shows a downward trend from the Triassic to the Jurassic with the highest peak in the Late Triassic and an increase in the Cretaceous

The Mann–Kendall test of the septal slope factors showed that there is no constant upward or downward trend within the Mesozoic data (tau = − 0.112, two-sided *p*-value = 0.46; Fig. 12). Since an increase during the end of the Triassic and in the Early Jurassic as well as a Cretaceous decrease was expected, the resulting *p*-values confirm the expectations. This corroborates the hypothesis that there is no monotonic trend.

## Discussion

### Palaeoenvironment—sea surface pH and pCO_2_

It has been suggested that an excessive build-up of CO_2_ in the atmosphere at the end of the Triassic caused an undersaturation of seawater with respect to CaCO_3_, which suppressed deposition of aragonite, high-Mg calcite, and to a lesser extend low-Mg calcite (Greene et al., 2012; Hautmann, 2004; Hautmann et al., 2008). Under these conditions, biocalcifying organisms with aragonitic or high-Mg calcitic skeletons were in a competitive disadvantage compared with those that either secreted less soluble low-Mg calcite or were non-calcifying (Cohen & Holcomb, 2009; Hautmann, 2004; Ries, 2005; Ries et al., 2006). A similar scenario can be assumed for ammonoids, with a competitive advantage of such families that invested less energy in the secretion of their thinner septa. We, therefore, hypothesised that ammonoids with thinner septa had a higher likelihood to survive the time of reduced seawater pH during the Triassic–Jurassic transition.

**Fig. 13:**
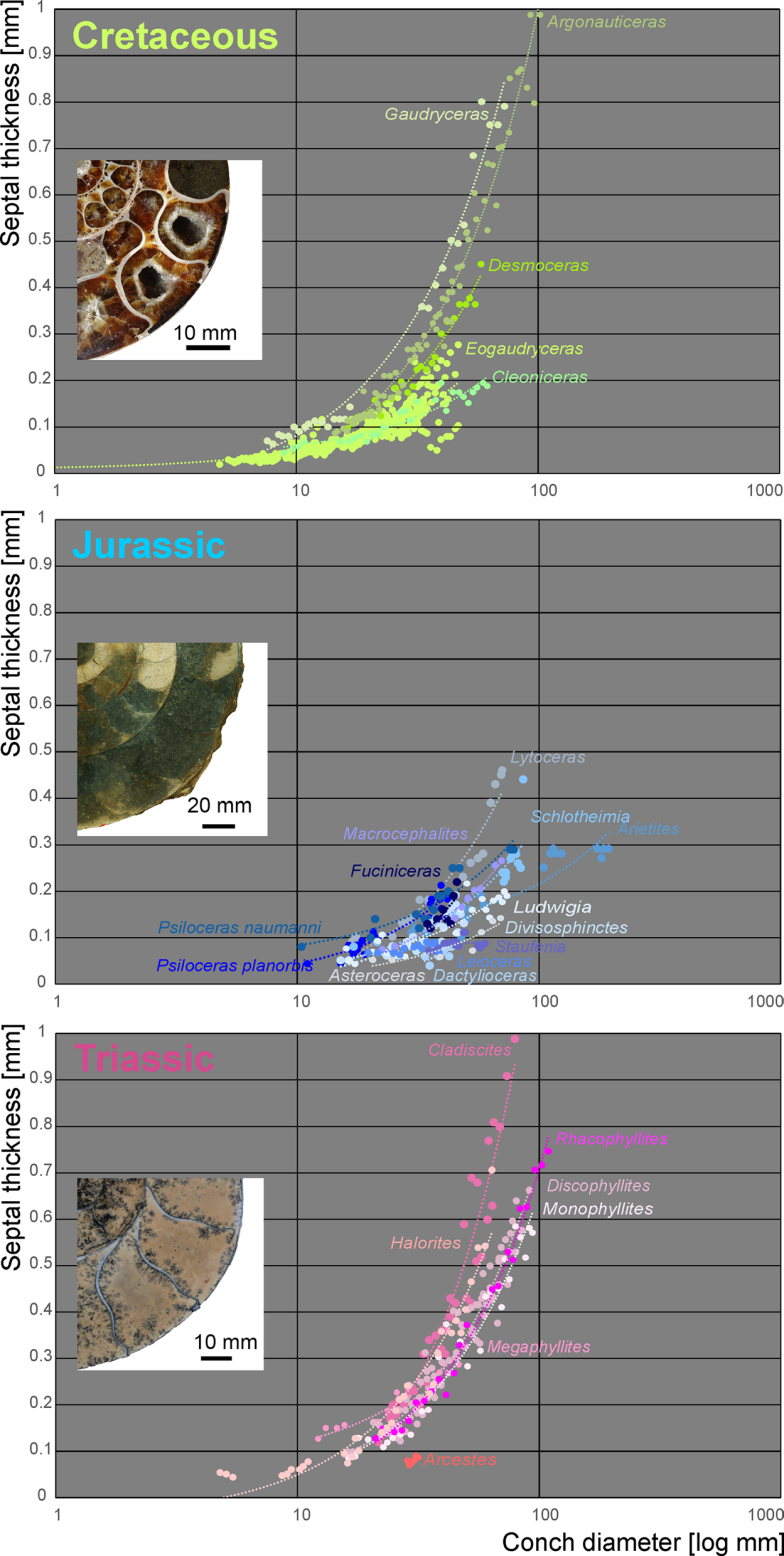
Conch diameter at the measured septum as a function of septal thickness grouped into the corresponding geological age. The dashed lines indicate the linear regression lines. Longitudinal sections of representative specimens illustrate the respective septal thickness. Jurassic: *Arietites* sp. (Arietitidae), Triassic: *Rhacophyllites neojurensi*s (Phylloceratidae), Cretaceous: *Argonauticeras besairei* (Lytoceratidae)

The ammonite septal slope factor, however, does not correlate significantly with raised atmospheric CO_2_- and declining pH-level around the Triassic–Jurassic transition (Figs. 10, 13). Nevertheless, the septal slope factor changed﻿ through time, from thick septa in the Late Triassic via thinner septa throughout the Jurassic back to thicker septa in the Early Cretaceous. Presuming that CO_2_- and declining pH-levels did influence the formation of ammonoid septa like other calcifying organisms (e.g., Hautmann, 2004; Orr et al., 2005), the question arises how would this become visible. As evident from our data, the septal slope factor differs between ammonoid clades, implying different strategies in coping with changes of hydrospheric composition. In other words, different groups of ammonoids invested different amounts of their energy into forming their septa. In turn, we can expect differences in the reaction to phases of increase oceanic CO_2_- and pH-levels. Similarly, a positive selection for ammonoids with thinner septa appears likely, even if only slight. If this assumption holds true, we can predict to find a decrease in septal slope factor following strong declines in oceanic pH-levels. This is what we found in our data. The absence of a strong rebound with taxa with thicker septa during the Jurassic might root in the fact that pH stays moderate during the Jurassic.

### Palaeoenvironment—seawater Mg/Ca ratio

A comparison between the septal slope factor and times of aragonite versus calcite sea conditions did not reveal a statistically significant correlation (Spearman’s rank correlation test; rho = − 0.001, two-sided *p*-value = 0.99) between septal thickness and phases of aragonite versus calcite seas. The analysis of this factor is hampered by the fact that sharp peaks of the septal slope factors during the Jurassic occur in some groups only. However, Fig. 10D shows that the Mg/Ca ratio declined at the Triassic–Jurassic boundary (Demicco et al., 2005) and consequently, calcite sea conditions prevailed thereafter (Stanley & Hardie, 1998).

Kiessling et al. (2008) suggest that Mg/Ca ratios of global oceans were less important for regulating long-term patterns of skeletal mineralogy than mass extinctions. In addition, they suggest that recovery from mass extinctions is more important than selective extinction in driving the Phanerozoic pattern of skeletal mineralogy. Perhaps, the physiological control of biomineralisation is too strong to be affected by Mg/Ca ratios, or the timescale of the Mg/Ca ratio fluctuations may be too slow to impose a strong selective pressure (Kiessling et al., 2008). In any case, our data (Fig. 10) do not provide evidence that Mg/Ca ratios and aragonite/calcite sea conditions influenced ammonoid septal thickness.

### The role of palaeolatitude

The geographical analysis in Fig. 9E–H documents septal thickness relative to diameter of the sampled ammonite species from their respective palaeolatitudes. Variation in thickness of biogenic calcareous structures was investigated by Watson et al. (2012); they described taxonomically controlled latitudinal variations in shell morphology and composition in various marine calcifiers. They found different patterns of shell thickness in various organisms. For example, shells or skeletal walls were thinner at higher latitudes and low temperatures in buccinid gastropods and in echinoids, whereas there was no such trend in brachiopods. While Watson et al. (2012) compared species of different invertebrate groups, we compared the variation in septal slope factor exclusively for ammonoids and found a correlation with palaeolatitude (Fig. 9F–H), indicating that ammonoids from higher latitudes had thinner septal thicknesses. When comparing average septal slope factors with palaeolatitude (without the outlier Divisosphinctes), the correlation is with *p* = 0.046 significant. This correlation also supports an impact of ocean acidification conditions on the relative septal thickness: According to Andersson et al. (2008), the effect of ocean acidification is greater in high latitudes because cool seawater is less supersaturated with respect to CaCO_3_ than warm seawater in lower latitudes. It is, therefore, reasonable to assume that ammonoids that lived in higher latitudes were more strongly affected by the end-Triassic ocean acidification event and had a higher selection pressure for thinner septa than ammonoids from lower latitudes.

Thin-shelled ammonoids (especially juvenile and subadult stages) are predicted to have suffered from these acidic conditions particularly, which might even have been lethal to some. Accordingly, we hypothesise that ammonoids that lived in higher latitudes were adapted to some degree to the decreased saturation state with respect to CaCO_3_ in higher latitudes and colder water. Nevertheless, the degree of reduction of septal thickness in boreal latitudes is low and should be interpreted with some reservation. The weakness of this signal might be rooted in the fact that the sample is small and that it comprises more ammonoids from Europe than from other continents. Thus, there might be biases from their phylogenetic position or their geographic origin.

### Phenotypic plasticity

Since our sampling includes ammonoids from different habitats with different environmental conditions, the variation in septal thickness could also be due to phenotypic plasticity (Agrawal, 2001; Monnet et al., 2015; West-Eberhard, 2003). This factor should optimally be considered when studying ecophenotypic and geographic variation (De Baets et al., 2015). Thus, the palaeolatitudinal differences in septal thickness between the families might reflect phenotypic plasticity due to different influences of environmental conditions in the habitat. The available data, however, do not allow us to conclude whether some ammonoids have thinner septa as result of phenotypic plasticity or of other evolutionary processes. In general, the assessment of effects of phenotypic plasticity in palaeontological data is hampered by the absence of genomic data in order to differentiate between a genetic and a purely phenotypic variation.

### Phylogeny

Apart from environmental effects, differences in septal thickness between members of certain ammonoid groups may reflect phylogenetic relationships. It is possible that species of different ammonoid lineages inherited different capabilities to cope with differences in sea water pH or simply produced septa of different thicknesses for other reasons (e.g., preferred water depth). Therefore, it was tested if each species has a specific septal thickness-to-diameter ratio and resulting septal slope factor and if these values also correspond with the systematic affiliation (on family level).

Our data reveal that each group of closely related species has their particular range in septal slope factor and intercept of mean septal thickness in relation to conch diameter (Figs. 8, 11, 13; Tables 1, 2, 5). Also, more closely related taxa appear to have more similar septal thicknesses (Table 5), but there are also overlaps between systematic groups. This suggests that the septal thickness is linked to some degree with the systematic position and the respective evolutionary history. Since ammonoids show quite high evolutionary rates (Sandoval et al., 2001), however, they might have adapted relatively quickly to changing ocean chemistry.

The question arises whether ammonoids were positively selected for such lineages with thinner septa during ocean acidification events. To address this question, we tested the temporal distribution of septal slope factors around the ocean acidification phase in the latest Triassic. Figure 12 shows the results of the Mann–Kendall Trend test for the relationship between stratigraphic position and septal thickness. The results of the test confirm that there is no monotonic trend in the data. This implies that there is a fluctuating trend within the data with the lowest values in the Early Jurassic. In turn, this corroborates the hypothesis that the sampled ammonoid lineages had thicker septa during much of the Triassic and Cretaceous while Jurassic ammonoid lineages had thinner septa relative to diameter (Figs. 10, 11, 12, 13, Table 6). This further implies that a positive selection for the groups that were more tolerant towards ocean acidification might have occurred (e.g., taxa better capable of physiological buffering; Eichenseer et al., 2019) in the course of the mass extinction at the end of the Triassic (Hallam, 2002). These groups could have adapted to the more acidic conditions by producing thinner septa in order to lower their energetic costs, slowly returning to forms with thicker septa later in the Jurassic.

Members of the Phylloceratidae are considered to be the only survivors of the end-Triassic mass extinction (Fig. 11) and to have given rise to all later ammonoid families via the Psiloceratidae (Page, 2008). The linear regression model shows that members of the family Lytoceratidae have the thickest septa relative to diameter and are closely followed by the family Phylloceratidae. This supports the hypothesis that septal thickness is to some degree phylogenetically controlled. The sampled Phylloceratidae are of Late Triassic age while most samples of the Lytoceratidae are from the Early Cretaceous; both show a similar ratio between septal thickness and diameter. When the septal slope factors of these two families are compared with those of the Arietitidae, Psiloceratidae and the only Early Jurassic lytoceratid, it turned out that Early Jurassic representatives of these lineages have thinner septa relative to diameter than their relatives from the Cretaceous and Triassic. The Jurassic pattern of septal thickness could therefore represent the phylogenetic preservation of the evolutionary processes that took place during the T–J ocean acidification event.

### Reduced septal thickness and its trade-offs

The function of septal frilling and thus the factors causing a selection for ever more complex septal frilling has been widely discussed (e.g., Hewitt & Westermann, 1987; Klug & Hoffmann, 2015; Klug et al., 2008; Lemanis, 2020; Peterman et al., 2021). These authors suggested, among other things, that septal frilling served for purposes such as liquid retention, increased growth rates, improved resistance against point loads (predation), as well as resistance against hydrostatic pressure. We do not know which of these factors were the most important and whether they are mutually exclusive or played roles of varying importance. Nevertheless, we agree with these authors that the overall evolutionary trend towards more complex septa is important and rather pervasive across most ammonoid lineages (e.g., Boyajian & Lutz, 1992; Klug & Hoffmann, 2015; Pérez-Claros & Bengtson, 2018; Saunders et al., 1999). In turn, a reduced septal thickness might have its negative effects by reducing stability. We do lack the data to test this. However, the reduction of septal thickness may have represented a factor causing negative selection in some lineages. This may explain, why the emerging picture of distribution of septal slope factors over lineages, through time and across latitudes became blurred: For some groups of ammonoids, the energetic costs to produce thicker and thus more resistant septa may have been of lesser importance than the mechanical strength they provided.

## Conclusions

Hypotheses for the causes and impacts of predicted future-relevant stressors on marine ecosystems such as ocean acidification can be tested using data of past long-term shifts in ocean water chemistry. In this study, relative septal thicknesses of Mesozoic ammonoids were measured to evaluate their potential value as palaeoecological proxy. We compared the distribution in time and space of septal thickness patterns with factors affecting seawater chemistry.

The ontogenetic trajectories of septal thickness were documented for 57 specimens. The relative septal thickness turned out to grow rather linearly throughout the ontogeny of individuals. By contrast, it differed significantly between lineages, through the Mesozoic, and depending on their palaeolatitudinal origins. Cretaceous members of the Lytoceratidae formed the thickest septa, followed by Triassic Cladiscitidae, Cretaceous Desmoceratidae and Triassic Phylloceratidae. Early Jurassic Arietitidae and Asteroceratidae produced the thinnest septa. The timing of the origin of these lineages implies a possible impact of ocean acidification on ammonoid evolution because those lineages with the thinnest septa diversified in the wake of the end-Triassic ocean acidification event. Nevertheless, we did not find a significant correlation with long-term atmospheric CO_2_ and sea surface pH.

Alternatively, a variation among the families and species with respect to septal thickness might also be explained partially by differences in phenotypic plasticity (Gause, 1947) of septal thickness. Phenotypic plasticity in relation to various environmental factors such as water energy and specialised predators is common in cephalopods (Boyle & Boletzky, 1996) and has also been suggested for Cretaceous ammonoids (Kin, 2010).

The measured palaeolatitudinal differences in septal thickness between the families would be in accordance with a stronger effect of ocean acidification in higher latitudes, but it could also reflect phenotypic plasticity controlled by differences in environmental conditions in the habitat. More data are needed to test these alternative hypotheses.

These results underline that septal thickness, chamber volumes and septal spacing of ammonoids can provide valuable palaeoenvironmental proxies. In order to increase the usefulness of these proxies, septal thickness and septal spacing of additional taxa and specimens should be measured with a high stratigraphic resolution to improve our understanding of the respective roles of phylogenetic signals and ocean chemistry. We consider these parameters as promising and hope that this study stimulates further research.

## Supplementary Information

**Additional file 1.** Excel sheet with all raw measurements of all ammonoids included and listed in Table 1.

**Additional file 2.** Word file with the R code used.

## Data Availability

The authors declare that all data supporting the findings of this study are available within this article and as supplementary files: an excel file with the raw measurements and the R code are available at Additional files 1 and 2.
