# Supplementary material for: Is the relative thickness of ammonoid septa influenced by ocean acidification, phylogenetic relationships and palaeogeographic position?
Source: Swiss J Palaeontol. 2022 Apr 18;141(1):4. doi: 10.1186/s13358-022-00246-2 (PMC9016059; doi:10.1186/s13358-022-00246-2)
Supplement: Supplementary file 2 — Additional file 2. Word file with the R code used. [file 13358_2022_246_MOESM2_ESM.docx]

**R Codes**

**of**

**Relative thickness of ammonoid septa influenced by ocean acidification, phylogeny and palaeogeographic position**

**by**

**Céline Weber, Michael Hautmann, Amane Tajika & Christian Klug**

**Code slope factor calculation**

### Discophyllites ebneri MEAN (A1-A5)

xA1bisA5<-c(7.243385, 6.733112, 6.219224, 5.875658, 5.553809, 5.232470, 4.819672, 4.483789, 3.567419, 3.350935, 3.202154, 2.936897, 2.789036, 2.598781, 2.523578, 2.189943, 7.983708, 7.551566, 6.917250, 6.213171, 5.735805, 5.189178, 4.743932, 3.725294, 3.399772, 3.166971, 2.873534, 2.605128, 2.045313, 7.921502, 7.353940, 6.976199, 6.674830, 6.391208, 6.019761, 5.632448, 5.263635, 4.899190, 4.583578, 4.382062, 4.179491, 3.900267, 3.657726, 2.898154, 2.770229, 2.644603, 2.495599, 2.327062, 2.170244, 9.032305, 8.392607, 7.704087, 7.202509, 6.757077, 6.280161, 5.825897, 5.455008, 3.933026, 3.660490, 3.425097, 3.262520, 3.060184, 2.768859, 2.533838, 6.771224, 6.262476, 5.813181, 5.444522, 5.062112, 4.782762, 4.506494, 4.207568, 3.900866, 3.602174, 3.407764, 3.238223, 3.097594, 2.646627, 2.479521, 2.326853, 1.777610, 1.676786, 1.604116)

yA1bisA5<- c(0.052913, 0.045635, 0.044904, 0.039006, 0.041078, 0.034735, 0.032276, 0.029986, 0.027912, 0.030787, 0.024793, 0.02024, 0.021039, 0.015345, 0.014504, 0.013367, 0.063665, 0.05691, 0.053588, 0.049674, 0.042568, 0.0407, 0.037247, 0.024819, 0.026948, 0.021166, 0.023798, 0.018751, 0.016191, 0.052597, 0.045617, 0.051273, 0.037038, 0.042698, 0.041544, 0.032887, 0.035888, 0.032199, 0.039691, 0.040168, 0.02657, 0.024004, 0.026408, 0.016828, 0.018675, 0.012157, 0.014672, 0.013558, 0.015475, 0.066037, 0.057379, 0.0590613, 0.055346, 0.050412, 0.042344, 0.043771, 0.033661, 0.028934, 0.021465, 0.021398, 0.017756, 0.018575, 0.014042, 0.01273, 0.051343, 0.043238, 0.047998, 0.041258, 0.040294, 0.036705, 0.030856, 0.029042, 0.024257, 0.022048, 0.02567, 0.018338, 0.017978, 0.015861, 0.01363, 0.014514, 0.009953, 0.009602, 0.009422)

coef(lm(yA1bisA5~xA1bisA5))

geom_abline(intercept = -0.0311, slope = 0.0077)

### Eogaudryceras umbilicostriatus MEAN (B1-B17)

xB1bisB17<-c(4.031295, 3.857879, 3.753551, 3.62288, 3.4711, 3.36709, 3.285049, 3.145608, 3.059917, 2.929603, 2.036268, 1.967979, 1.905489, 1.852993, 1.818682, 1.762871, 1.704275, 4.049859, 3.489952, 3.365972, 3.163929, 3.03136, 2.746336, 2.637822, 1.776743,

1.47204, 1.445919, 3.200717, 3.138017, 2.993149, 2.91447, 2.811113, 2.751744, 2.587651, 2.530934, 2.46082, 2.132036, 2.05336, 1.974459, 1.74741, 1.70469, 1.637016, 1.580932,

1.533941, 1.480325, 1.436701, 1.391875, 1.351506, 1.211428, 1.167879, 1.127213, 1.077684, 1.045113, 0.88295, 0.852621, 0.811053, 3.081736, 2.918924, 2.811429, 2.427608, 2.342491, 2.266862, 2.148757, 2.077618, 1.989607, 1.929645, 1.855064, 1.806193, 1.739169, 1.720041, 1.312086, 1.267196, 1.219887, 1.18008, 1.132466, 1.098918, 1.061043, 1.030301, 0.983718, 0.930825, 0.687339, 0.66964, 0.643992, 0.618661, 0.593979, 0.571793, 4.146257, 4.232832, 4.064472, 3.878638, 2.746336, 2.665015, 2.597572, 2.524274, 2.451145, 2.395594, 2.315989, 1.948184, 1.892005, 3.914026, 3.800406, 3.628161, 3.497333, 3.376486, 3.291139, 3.166729, 3.062749, 2.938032, 2.862501, 2.742489, 2.674901, 2.605229, 2.49962, 2.106698, 2.039361, 1.980301, 1.923104, 1.507085, 1.469277, 1.431166, 1.119255, 1.083113, 1.042176, 0.817521, 0.797882, 0.757288, 0.576423, 0.552875, 3.50422, 3.359213, 3.265914, 3.163205, 3.056726, 2.972134, 2.908936, 2.821738, 2.734328, 2.648874, 2.554455, 2.464614, 1.0684, 1.037153, 0.994033, 0.953978, 2.621766, 2.552697, 2.430267, 2.354728, 2.268504, 2.205409, 2.16532, 2.09885, 2.008203, 1.477914, 1.441122, 1.396767, 1.332879, 1.298103, 1.255942, 1.217774, 1.186333, 1.127105, 1.084525, 0.806051, 0.789646, 0.75795, 0.728502, 0.707495, 0.676627, 0.647758, 3.063392, 2.96945, 2.876312, 2.786811, 2.316686, 2.248366, 2.207476, 2.117761, 1.698613, 1.62528, 1.574361, 1.330939, 1.284021, 0.930367, 0.902203, 0.857402, 0.48015, 3.239899, 3.145696, 3.076834, 2.996359, 2.897697, 2.808338, 2.720348, 2.632547, 2.519709, 2.405821, 2.341978, 2.240385, 2.160717, 1.686562, 1.645951, 1.589877, 1.537794, 1.457058, 1.416165, 1.328098, 1.18018, 1.144643, 1.09448, 1.053863, 0.70689, 0.673033, 0.656106, 0.627906, 0.607079, 0.574404, 0.539258, 0.521557, 3.082355, 2.955909, 2.377471, 2.305506, 2.263304, 2.186918, 2.107947, 2.036404, 1.976742, 1.897654, 1.854927, 1.799364, 1.732675, 1.658786, 1.202144, 1.154731, 1.124815, 1.08327, 1.043862, 2.564622, 2.494018, 2.375398, 2.321368, 2.259943, 2.207533, 2.1312, 2.051296, 1.986006, 1.914555, 1.841941, 1.686856, 1.670414, 1.591188, 1.551332, 1.487687, 1.411173, 1.354673, 1.311265, 1.275937, 0.882484, 0.83401, 0.810616, 0.777712, 0.752654, 0.725597, 0.693718, 0.675902, 3.179908, 3.033589, 2.956726, 2.85259, 2.766651, 2.683695, 2.580808, 2.473637, 2.13336, 2.067615, 2.030513, 1.97046, 1.922809, 1.458652, 1.404363, 1.350639, 1.303877, 1.252664, 1.2035, 1.164812, 1.122289, 0.827665, 0.737269, 0.705462, 0.68056, 0.656659, 3.031621, 2.945944, 2.845454, 2.35299, 2.284267, 2.225204, 2.136548, 2.082942, 2.033054, 1.950429, 1.876123, 1.796856, 1.723201, 1.661808, 1.613854, 1.563396, 1.196339, 1.132446, 1.118045, 1.067276, 1.013617, 3.046131, 2.915993, 2.826606, 2.752757, 2.668048, 2.590171, 2.514323, 2.454618, 2.372092, 1.976877, 1.926675, 1.874629, 1.819878, 1.767784, 1.730912, 1.650969, 1.592701, 1.531942, 1.46951, 1.425976, 1.36507, 0.903673, 0.818512, 2.292086, 2.230442, 2.121391, 1.751969, 1.692604, 1.274089, 1.221148, 1.171466, 1.122167, 1.083137, 1.045598, 1.010829, 0.980187, 0.942581, 4.674804, 4.542022, 4.339578, 4.193523, 3.962767, 3.81218, 3.595118, 3.470102, 3.348419, 3.206135, 3.124122, 3.019367, 2.855658, 2.107439, 2.03235, 1.968476, 1.880711, 1.62117, 1.165738, 1.128249)

yB1bisB17<- c(0.021135, 0.019552, 0.017726, 0.015541, 0.014255, 0.015359, 0.014403, 0.013876, 0.013152, 0.012707, 0.009109, 0.008133, 0.007837, 0.008857, 0.006777, 0.006338, 0.006046, 0.017418, 0.016953, 0.013546, 0.013675, 0.011454, 0.011832, 0.011074, 0.007433, 0.007564, 0.00708, 0.014663, 0.014314, 0.014281, 0.015235, 0.011209, 0.010211, 0.01171, 0.010416, 0.011984, 0.010497, 0.008988, 0.009925, 0.007949, 0.006341, 0.006128, 0.006087, 0.006349, 0.006716, 0.005805, 0.006572, 0.005668, 0.005114, 0.005006, 0.005421, 0.005136, 0.005136, 0.005082, 0.004017, 0.004193, 0.01645, 0.01505, 0.015239, 0.011202, 0.009881, 0.009619, 0.011776, 0.011826, 0.009715, 0.009499, 0.009138, 0.00895, 0.008706, 0.008152, 0.006374, 0.005889, 0.006489, 0.00673, 0.006379, 0.005524, 0.00641, 0.005418, 0.003721, 0.004998, 0.003759, 0.003472, 0.00371, 0.00359, 0.003513, 0.003252, 0.01993, 0.019122, 0.019227, 0.019576 0.009393, 0.009449, 0.0107, 0.008931, 0.009716, 0.009298, 0.009124, 0.007145 0.007312, 0.020899, 0.020665, 0.019659, 0.019002, 0.017058, 0.016228, 0.015512, 0.014757, 0.013626, 0.012904, 0.012078, 0.012935, 0.011613 0.010608, 0.012856, 0.009374, 0.011598, 0.007628, 0.006952, 0.006558 0.006406, 0.005475, 0.00454, 0.004166, 0.004402, 0.003256, 0.003275, 0.002888, 0.002449, 0.016524, 0.018469, 0.015821, 0.015404, 0.013713, 0.012003, 0.012437, 0.011264, 0.010245, 0.011815, 0.010776, 0.011501, 0.007727, 0.007066, 0.006916, 0.006905, 0.011398, 0.011539, 0.010503, 0.009297, 0.008807, 0.009068, 0.008691, 0.008093, 0.009132, 0.006688, 0.00765, 0.007615, 0.005715, 0.005888, 0.005653, 0.006545, 0.005507, 0.005175, 0.00503, 0.003412, 0.003332, 0.00337, 0.003546, 0.003757, 0.003344, 0.002923, 0.012717, 0.011977, 0.010525, 0.012396, 0.009148, 0.008692, 0.008028, 0.007901, 0.006837, 0.008155, 0.005992, 0.004937, 0.005084, 0.00389, 0.004598, 0.004555, 0.002347, 0.024101, 0.013823, 0.012651, 0.012719, 0.012597, 0.010926, 0.011788, 0.011658, 0.010808, 0.010755, 0.008981, 0.009687, 0.009712, 0.008169, 0.007267, 0.007408, 0.006886, 0.007321, 0.00729, 0.005462, 0.006209, 0.005163, 0.004933, 0.005826, 0.003727, 0.003455, 0.003901, 0.003212, 0.003359, 0.003225, 0.003086, 0.003151, 0.013326, 0.013685, 0.01159, 0.011549, 0.008306, 0.009844, 0.010039, 0.008642, 0.009477, 0.008212, 0.00892, 0.008644, 0.007471, 0.006663, 0.00709, 0.005794, 0.004953, 0.004992, 0.00502, 0.010806, 0.011245, 0.010842, 0.009061, 0.009505, 0.00968, 0.008933, 0.008875, 0.009157, 0.009584, 0.008956, 0.00703, 0.007284, 0.006872, 0.006394, 0.007228, 0.00649, 0.006602, 0.006259, 0.005808, 0.004539, 0.003889, 0.004029, 0.00374, 0.00399, 0.004285, 0.00388, 0.00385,

0.015428, 0.013692, 0.013134, 0.012876, 0.01255, 0.012394, 0.011753, 0.010759, 0.009461, 0.010713, 0.009512, 0.009036, 0.008097, 0.006683, 0.006058, 0.00562, 0.004488, 0.004914, 0.004657, 0.00563, 0.005241, 0.004082, 0.002852, 0.002618, 0.003191, 0.002861, 0.01317, 0.014201, 0.014732, 0.013836, 0.013342, 0.0128, 0.009743, 0.009554,

0.009131, 0.007987, 0.0083977, 0.008146, 0.00742, 0.007856, 0.007037, 0.006705, 0.006307, 0.006393, 0.005493, 0.005584, 0.00497, 0.012154, 0.01058, 0.012149, 0.011363, 0.011956, 0.011681, 0.010503, 0.010234, 0.008795, 0.007813, 0.008843, 0.007289, 0.006899, 0.00729, 0.007416, 0.007182, 0.006499, 0.006413, 0.006332, 0.006535, 0.005576, 0.003697, 0.003284, 0.01081, 0.01001, 0.008064, 0.008358, 0.007641, 0.005512, 0.00614, 0.005099, 0.005338, 0.005256, 0.004506, 0.003932, 0.003239, 0.003728, 0.028443, 0.0215564, 0.026179, 0.023954, 0.024504, 0.022805, 0.021197, 0.020007, 0.016142, 0.015716, 0.014989, 0.015638, 0.015363, 0.010151, 0.01264, 0.010854, 0.009826, 0.00867, 0.005957, 0.006026)

coef(lm(yB1bisB17~xB1bisB17))

geom_abline(intercept = 0.01218, slope = 0.004)

### Argonauticeras besairei MEAN (B19-B24)

xB19bisB24<-c(10.230444, 9.339951, 8.500612, 7.671929, 7.147225, 6.522221, 6.040766, 5.488527, 5.172479, 4.761607, 4.443403, 4.205346, 3.815032, 3.479031, 3.28958, 3.117327,

2.907504, 2.180317, 2.056563, 1.917293, 1.833872, 1.738494, 1.641248, 1.576252, 1.482171, 6.751524, 6.197564, 5.567706, 5.064138, 4.667205, 4.294048, 4.046834, 3.791045, 2.981165, 2.776047, 2.660343, 2.473536, 2.317297, 2.221775, 2.109501, 2.001097, 1.876131, 1.790612, 10.413173, 9.665816, 9.034221, 8.283902, 7.610921, 6.892558, 6.20551, 4.385229, 4.104272, 2.202499, 2.13545, 5.109867, 4.863846, 4.430677, 4.205134, 3.926756, 3.683659, 3.398057, 3.197058, 3.061784, 2.590629, 2.452005, 2.319031, 1.909947, 1.831567, 1.742128, 1.631549, 1.454904, 8.905203, 7.754479, 6.917362, 6.571929, 6.249435, 5.83851, 3.21951, 3.116935, 2.9186, 2.812293, 2.6299, 2.609918, 2.491549, 2.355967, 1.802714, 1.753603, 1.694617, 1.585452, 1.495173, 1.417804, 1.319242, 1.231964, 1.150354, 1.083444, 1.011657, 0.940815, 5.983942, 5.500043, 5.124493, 3.403019, 3.259655, 3.054922, 2.83356, 2.723196, 2.122223, 1.979792, 1.848507, 1.736089, 1.626088, 1.534803)

yB19bisB24<- c(0.098968, 0.099024, 0.087105, 0.085054, 0.070267, 0.066855, 0.058908, 0.060461, 0.050607, 0.040715, 0.039196, 0.033366, 0.031122, 0.027822, 0.026674, 0.025202, 0.026866, 0.01528, 0.013379, 0.013173, 0.012175, 0.011713, 0.011766, 0.010095, 0.009396, 0.057804, 0.052488, 0.054963, 0.052205, 0.04441, 0.039397, 0.037957, 0.034019, 0.024607, 0.019086, 0.017887, 0.016928, 0.017317, 0.014169, 0.01291, 0.012473, 0.012196, 0.012104, 0.103842, 0.079841, 0.083122, 0.08677, 0.073554, 0.070269, 0.067128, 0.033122, 0.027586, 0.016824, 0.017537, 0.052243, 0.045207, 0.041726, 0.037594, 0.034596, 0.032398, 0.030019, 0.027723, 0.027023, 0.021865, 0.021446, 0.021344, 0.017833, 0.012336, 0.010766, 0.00925, 0.008916, 0.079878, 0.064452, 0.072215, 0.066709, 0.062064, 0.053341, 0.024608, 0.022664, 0.020532, 0.019528, 0.018406, 0.019404, 0.016492, 0.01512, 0.010816, 0.009514, 0.00761, 0.008625, 0.007433, 0.007629, 0.006986, 0.007634, 0.007697, 0.007277, 0.007037, 0.006266, 0.060318, 0.053035, 0.045453, 0.028414, 0.022907, 0.020298, 0.019564, 0.019256, 0.016141, 0.014118, 0.01259, 0.012045, 0.008849, 0.010265)

coef(lm(yB19bisB24~xB19bisB24))

geom_abline(intercept = -0.0706, slope = 0.0107)

### Ludwigia bradfordiensis MEAN (E1 & E3)

xE1undE3<- c(3.293848, 3.073083, 2.925333, 2.777578, 2.603345, 2.470298, 2.35894, 2.256705, 2.149469, 7.725651, 7.542721, 7.39379, 7.147522, 6.788121, 6.551938, 6.328132, 6.113061, 5.548297, 5.280439, 4.98145, 4.755452, 2.710533, 2.574784, 1.675631)

yE1undE3<- c(0.007826, 0.006328, 0.005331, 0.006036, 0.006351, 0.005497, 0.009365, 0.008799, 0.005633, 0.016539, 0.020912, 0.016392, 0.016658, 0.020231, 0.013346, 0.013964, 0.016602, 0.014028, 0.018842, 0.017901, 0.012462, 0.005966, 0.00577, 0.006218)

coef(lm(yE1undE3~xE1undE3))

geom_abline(intercept = 0.005, slope = 0.0024)

### Staufenia opalinoides MEAN (E2 & E4)

coef(lm(yE2undE4~xE2undE4))

geom_abline(intercept = 0.0689, slope = 0.0004)

### Cladiscites sp. MEAN (F2-F3)

coef(lm(yF2undF3~xF2undF3))

geom_abline(intercept = -0.1953, slope =0.0147)

### Divisosphinctes besairei MEAN (O1-O2)

coef(lm(yO1und=2~xO1undO2))

geom_abline(intercept = -0.0191, slope = 0.0037

### Arietites sp.

coef(lm(yJ1bisJ2~xJ1bisJ2))

geom_abline(intercept = 0.0973, slope = 0.0012)

### Psiloceras planorbis MEAN (M1-M3)

coef(lm(yM1bisM3~xM1bisM3))

geom_abline(intercept = -0.0064, slope = 0.0047)

### Megaphyllites sp.

coef(lm(yA6~xA6))

geom_abline(intercept = 0.0647, slope = 0.0054)

### Monophyllites sp.

coef(lm(yA7~xA7))

geom_abline(intercept = -0.0206, slope = 0.0067)

### Rhacophyllites neojurensis

coef(lm(yA8~xA8))

geom_abline(intercept = -0.0433, slope = 0.0075)

### Gaudryceras sp.

coef(lm(yB18~xB18))

geom_abline(intercept = -0.0445, slope = 0.0124)

#### Lytoceras fimbriatum

coef(lm(yB25~xB25))

geom_abline(intercept = -0.0528, slope = 0.0067)

#### Desmoceras sp.

coef(lm(yC1~xC1))

geom_abline(intercept = -0.0345, slope = 0.0078)

### Cleoniceras sp.

coef(lm(yD1~xD1))

geom_abline(intercept = -0.0237, slope = 0.0028

### Leioceras sp.

coef(lm(yE5~xE5))

geom_abline(intercept = 0.0451, slope = 0.001)

### Halorites sp.

coef(lm(yF1~xF1))

geom_abline(intercept = -0.0519, slope = 0.0098)

### Arcestes sp.

coef(lm(yG1~xG1))

geom_abline(intercept = -0.0639, slope = 0.0048)

### Macrocephalites compressus

coef(lm(yH1~xH1))

geom_abline(intercept = -0.027, slope = 0.0041)

### Asteroceras sp.

coef(lm(yL1~xL1))

geom_abline(intercept = 0.0019, slope = 0.0019)

### Psiloceras naumanni

coef(lm(yM4~xM4))

geom_abline(intercept = 0.0499, slope = 0.0034)

### Schlotheimia sp.

coef(lm(yN1~xN1))

geom_abline(intercept = -0.0065, slope = 0.0036)

### Fuciniceras cf. isseli

coef(lm(yJ3~xJ3))

geom_abline(intercept = -0.0103, slope = 0.0064)

**Codes Figures**

**Codes Figures 8**

install.packages("ggplot2")

library(ggplot2)

library(readr)

Group.A <- read_delim("~/Desktop/Group A.csv", ";", escape_double = FALSE, trim_ws = TRUE)

Group.B <- read_delim("~/Desktop/Group B.csv", ";", escape_double = FALSE, trim_ws = TRUE)

Group.C <- read_delim("~/Desktop/Group C.csv", ";", escape_double = FALSE, trim_ws = TRUE)

Group.D <- read_delim("~/Desktop/Group D.csv", ";", escape_double = FALSE, trim_ws = TRUE)

Group.E <- read_delim("~/Desktop/Group E.csv", ";", escape_double = FALSE, trim_ws = TRUE)

Group.F <- read_delim("~/Desktop/Group F.csv", ";", escape_double = FALSE, trim_ws = TRUE)

Group.G <- read_delim("~/Desktop/Group G.csv", ";", escape_double = FALSE, trim_ws = TRUE)

Group.H <- read_delim("~/Desktop/Group H.csv", ";", escape_double = FALSE, trim_ws = TRUE)

Group.J <- read_delim("~/Desktop/Group J2.csv",";", escape_double = FALSE, trim_ws = TRUE)

Group.K <- read_delim("~/Desktop/Group K.csv",";", escape_double = FALSE, trim_ws = TRUE)

Group.L <- read_delim("~/Desktop/Group L.csv", ";", escape_double = FALSE, trim_ws = TRUE)

Group.M <- read_delim("~/Desktop/Group M2_new.csv", ";", escape_double = FALSE, trim_ws = TRUE)

Group.N <- read_delim("~/Desktop/Group N.csv",";", escape_double = FALSE, trim_ws = TRUE)

Group.O <- read_delim("~/Desktop/Group O.csv", ";", escape_double = FALSE, trim_ws = TRUE)

Group.P <- read_delim("~/Desktop/Group P.csv", ";", escape_double = FALSE, trim_ws = TRUE)

Group.A <- read.csv("~/Desktop/Group A.csv", header=TRUE, sep=";")

Group.B <- read.csv("~/Desktop/Group B.csv", header=TRUE, sep=";")

Group.C <- read.csv("~/Desktop/Group C.csv", header=TRUE, sep=";")

Group.D <- read.csv("~/Desktop/Group D.csv", header=TRUE, sep=";")

Group.E <- read.csv("~/Desktop/Group E.csv", header=TRUE, sep=";")

Group.F <- read.csv("~/Desktop/Group F.csv", header=TRUE, sep=";")

Group.G <- read.csv("~/Desktop/Group G.csv", header=TRUE, sep=";")

Group.H <- read.csv("~/Desktop/Group H.csv", header=TRUE, sep=";")

Group.J <- read.csv("~/Desktop/Group J2.csv", header=TRUE, sep=";")

Group.K <- read.csv("~/Desktop/Group K.csv", header=TRUE, sep=";")

Group.L <- read.csv("~/Desktop/Group L.csv", header=TRUE, sep=";")

Group.M <- read.csv("~/Desktop/Group M2.csv", header=TRUE, sep=";")

Group.N <- read.csv("~/Desktop/Group N.csv", header=TRUE, sep=";")

Group.O <- read.csv("~/Desktop/Group O.csv", header=TRUE, sep=";")

Group.P <- read.csv("~/Desktop/Group P.csv", header=TRUE, sep=";")

ggplot() +

geom_point(data=Group.A, aes(y=MeanSeptumA1, x=DiameterA1), color='blue')+

geom_point(data=Group.A, aes(y=MeanSeptumA2, x=DiameterA2), color='blue')+

geom_point(data=Group.A, aes(y=MeanSeptumA3, x=DiameterA3), color='blue')+

geom_point(data=Group.A, aes(y=MeanSeptumA4, x=DiameterA4), color='blue')+

geom_point(data=Group.A, aes(y=MeanSeptumA5, x=DiameterA5), color='blue')+

geom_point(data=Group.A, aes(y=MeanSeptumA6, x=DiameterA6), color='blue')+

geom_point(data=Group.A, aes(y=MeanSeptumA7, x=DiameterA7), color='blue')+

geom_point(data=Group.A, aes(y=MeanSeptumA8, x=DiameterA8), color='blue')+

geom_point(data=Group.B, aes(y=MeanSeptumB1, x=DiameterB1), color='blueviolet') +

geom_point(data=Group.B, aes(y=MeanSeptumB2, x=DiameterB2), color='blueviolet')+

geom_point(data=Group.B, aes(y=MeanSeptumB3, x=DiameterB3), color='blueviolet')+

geom_point(data=Group.B, aes(y=MeanSeptumB4, x=DiameterB4), color='blueviolet')+

geom_point(data=Group.B, aes(y=MeanSeptumB5, x=DiameterB5), color='blueviolet')+

geom_point(data=Group.B, aes(y=MeanSeptumB6, x=DiameterB6), color='blueviolet')+

geom_point(data=Group.B, aes(y=MeanSeptumB7, x=DiameterB7), color='blueviolet')+

geom_point(data=Group.B, aes(y=MeanSeptumB8, x=DiameterB8), color='blueviolet')+

geom_point(data=Group.B, aes(y=MeanSeptumB9, x=DiameterB9), color='blueviolet') +

geom_point(data=Group.B, aes(y=MeanSeptumB10, x=DiameterB10), color='blueviolet') +

geom_point(data=Group.B, aes(y=MeanSeptumB11, x=DiameterB11), color='blueviolet') +

geom_point(data=Group.B, aes(y=MeanSeptumB12, x=DiameterB12), color='blueviolet') +

geom_point(data=Group.B, aes(y=MeanSeptumB13, x=DiameterB13), color='blueviolet') +

geom_point(data=Group.B, aes(y=MeanSeptumB14, x=DiameterB14), color='blueviolet') +

geom_point(data=Group.B, aes(y=MeanSeptumB15, x=DiameterB15), color='blueviolet') +

geom_point(data=Group.B, aes(y=MeanSeptumB16, x=DiameterB16), color='blueviolet') +

geom_point(data=Group.B, aes(y=MeanSeptumB17, x=DiameterB17), color='blueviolet') +

geom_point(data=Group.B, aes(y=MeanSeptumB18, x=DiameterB18), color='blueviolet') +

geom_point(data=Group.B, aes(y=MeanSeptumB19, x=DiameterB19), color='blueviolet') +

geom_point(data=Group.B, aes(y=MeanSeptumB20, x=DiameterB20), color='blueviolet') +

geom_point(data=Group.B, aes(y=MeanSeptumB21, x=DiameterB21), color='blueviolet') +

geom_point(data=Group.B, aes(y=MeanSeptumB22, x=DiameterB22), color='blueviolet') +

geom_point(data=Group.B, aes(y=MeanSeptumB23, x=DiameterB23), color='blueviolet') +

geom_point(data=Group.B, aes(y=MeanSeptumB24, x=DiameterB24), color='blueviolet') +

geom_point(data=Group.B, aes(y=MeanSeptumB25, x=DiameterB25), color='blueviolet') +

geom_point(data=Group.C, aes(y=MeanSeptumC1, x=DiameterC1), color='orange')+

geom_point(data=Group.D, aes(y=MeanSeptumD1, x=DiameterD1), color='red')+

geom_point(data=Group.E, aes(y=MeanSeptumE1, x=DiameterE1), color='green')+

geom_point(data=Group.E, aes(y=MeanSeptumE2, x=DiameterE2), color='green')+

geom_point(data=Group.E, aes(y=MeanSeptumE3, x=DiameterE3), color='green')+

geom_point(data=Group.E, aes(y=MeanSeptumE4, x=DiameterE4), color='green')+

geom_point(data=Group.E, aes(y=MeanSeptumE5, x=DiameterE5), color='green')+

geom_point(data=Group.F, aes(y=MeanSeptumF1, x=DiameterF1), color='cyan')+

geom_point(data=Group.F, aes(y=MeanSeptumF2, x=DiameterF2), color='cyan')+

geom_point(data=Group.F, aes(y=MeanSeptumF3, x=DiameterF3), color='cyan')+

geom_point(data=Group.G, aes(y=MeanSeptumG1, x=DiameterG1), color='green4')+

geom_point(data=Group.H, aes(y=MeanSeptumH1, x=DiameterH1), color='darkorchid1')+

geom_point(data=Group.J, aes(y=MeanSeptumJ1, x=DiameterJ1), color='peru')+

geom_point(data=Group.J, aes(y=MeanSeptumJ2, x=DiameterJ2), color='peru')+

geom_point(data=Group.K, aes(y=MeanSeptumK1, x=DiameterK1), color='tomato1')+

geom_point(data=Group.L, aes(y=MeanSeptumL1, x=DiameterL1), color='seagreen3')+

geom_point(data=Group.M, aes(y=MeanSeptumM1, x=DiameterM1), color='deeppink2')+

geom_point(data=Group.M, aes(y=MeanSeptumM2, x=DiameterM2), color='deeppink2')+

geom_point(data=Group.M, aes(y=MeanSeptumM3, x=DiameterM3), color='deeppink2')+

geom_point(data=Group.M, aes(y=MeanSeptumM4, x=DiameterM4), color='deeppink2')+

geom_point(data=Group.N, aes(y=MeanSeptumN1, x=DiameterN1), color='yellow')+

geom_point(data=Group.O, aes(y=MeanSeptumO1, x=DiameterO1), color='darkgrey')+

geom_point(data=Group.O, aes(y=MeanSeptumO2, x=DiameterO2), color='darkgrey')+

geom_point(data=Group.P, aes(y=MeanSeptumP1, x=DiameterP1), color='black')+

geom_abline(intercept =xxx, slope = xxx, color='blue')+ #GroupA

geom_abline(intercept = xxx, slope = xxx, color='blueviolet')+ #GroupB

geom_abline(intercept = xxx, slope = xxx, color='orange')+ #GroupC

geom_abline(intercept = xxx, slope = xxx, color='red')+ #GroupD

geom_abline(intercept = xxx, slope = xxx, color='green')+ #GroupE

geom_abline(intercept = xxx, slope = xxx, color='cyan')+ #GroupF

geom_abline(intercept = xxx, slope = xxx, color= "green4")+ #GroupG

geom_abline(intercept = xxx, slope = xxx, color= "darkorchid1") #GroupH

geom_abline(intercept = xxx, slope = xxx, color='peru')+ #GroupJ

geom_abline(intercept =xxx, slope =xxx, color='tomato1')+ #GroupK

geom_abline(intercept =xxx, slope = xxx, color='seagreen3')+ #GroupL

geom_abline(intercept = xxx, slope = xxx, color='deeppink2')+ #GroupM

geom_abline(intercept =xxx, slope = xxx, color='yellow')+ #Group N

geom_abline(intercept =xxx, slope = xxx, color='darkgrey')+ #GroupO

geom_abline(intercept =xxx, slope = xxx, color='black')+ #Group P

last_plot() + xlab("Septa thickness [cm]")

last_plot() + ylab("Diameter [cm]")

last_plot() + theme_classic()

**Code Figure 10**

ggplot() +

geom_point(data=GroupA, aes(x=DiameterA1, y=MeanSeptumA1), color='white')+

geom_point(data=GroupA, aes(x=DiameterA2, y=MeanSeptumA2,), color='white')+

geom_point(data=GroupA, aes(x=DiameterA3, y=MeanSeptumA3,), color='white')+

geom_point(data=GroupA, aes(x=DiameterA4, y=MeanSeptumA4,), color='white')+

geom_point(data=GroupA, aes(x=DiameterA5, y=MeanSeptumA5,), color='white')+

geom_point(data=GroupA, aes(x=DiameterA6, y=MeanSeptumA6,), color='white')+

geom_point(data=GroupA, aes(x=DiameterA7, y=MeanSeptumA7,), color='white')+

geom_point(data=GroupA, aes(x=DiameterA8, y=MeanSeptumA8,), color='white')+

geom_abline(intercept =xxx, slope = xxx, col="red")+ #phyllos Triassic A

theme_classic()

last_plot() + xlab("Septa thickness [cm]")

last_plot() + ylab("Diameter [cm]")

last_plot() + ylab(0,10)

ggplot() +

geom_point(data=GroupB, aes(y=MeanSeptumB1, x=DiameterB1), color='white')+

geom_point(data=GroupB, aes(y=MeanSeptumB2, x=DiameterB2), color='white')+

geom_point(data=GroupB, aes(y=MeanSeptumB3, x=DiameterB3), color='white')+

geom_point(data=GroupB, aes(y=MeanSeptumB4, x=DiameterB4), color='white')+

geom_point(data=GroupB, aes(y=MeanSeptumB5, x=DiameterB5), color='white')+

geom_point(data=GroupB, aes(y=MeanSeptumB6, x=DiameterB6), color='white')+

geom_point(data=GroupB, aes(y=MeanSeptumB7, x=DiameterB7), color='white')+

geom_point(data=GroupB, aes(y=MeanSeptumB8, x=DiameterB8), color='white')+

geom_point(data=GroupB, aes(y=MeanSeptumB9, x=DiameterB9), color='white')+

geom_point(data=GroupB, aes(y=MeanSeptumB10, x=DiameterB10), color='white')+

geom_point(data=GroupB, aes(y=MeanSeptumB11, x=DiameterB11), color='white')+

geom_point(data=GroupB, aes(y=MeanSeptumB12, x=DiameterB12), color='white')+

geom_point(data=GroupB, aes(y=MeanSeptumB13, x=DiameterB13), color='white')+

geom_point(data=GroupB, aes(y=MeanSeptumB14, x=DiameterB14), color='white')+

geom_point(data=GroupB, aes(y=MeanSeptumB15, x=DiameterB15), color='white')+

geom_point(data=GroupB, aes(y=MeanSeptumB16, x=DiameterB16), color='white')+

geom_point(data=GroupB, aes(y=MeanSeptumB17, x=DiameterB17), color='white')+

geom_point(data=GroupB, aes(y=MeanSeptumB18, x=DiameterB18), color='white')+

geom_point(data=GroupB, aes(y=MeanSeptumB19, x=DiameterB19), color='white')+

geom_point(data=GroupB, aes(y=MeanSeptumB20, x=DiameterB20), color='white')+

geom_point(data=GroupB, aes(y=MeanSeptumB21, x=DiameterB21), color='white')+

geom_point(data=GroupB, aes(y=MeanSeptumB22, x=DiameterB22), color='white')+

geom_point(data=GroupB, aes(y=MeanSeptumB23, x=DiameterB23), color='white')+

geom_point(data=GroupB, aes(y=MeanSeptumB24, x=DiameterB24), color='white')+

geom_point(data=GroupB, aes(y=MeanSeptumB25, x=DiameterB25), color='white')+

geom_abline(intercept = xxx, slope = xxx, col="red")+ #L. fimbriantum

theme_classic()

last_plot() + xlab("Septa thickness [cm]")

last_plot() + ylab("Diameter [cm]")

ggplot() +

geom_point(data=GroupB, aes(y=MeanSeptumB1, x=DiameterB1), color='white')+

geom_point(data=GroupB, aes(y=MeanSeptumB2, x=DiameterB2), color='white')+

geom_point(data=GroupB, aes(y=MeanSeptumB3, x=DiameterB3), color='white')+

geom_point(data=GroupB, aes(y=MeanSeptumB4, x=DiameterB4), color='white')+

geom_point(data=GroupB, aes(y=MeanSeptumB5, x=DiameterB5), color='white')+

geom_point(data=GroupB, aes(y=MeanSeptumB6, x=DiameterB6), color='white')+

geom_point(data=GroupB, aes(y=MeanSeptumB7, x=DiameterB7), color='white')+

geom_point(data=GroupB, aes(y=MeanSeptumB8, x=DiameterB8), color='white')+

geom_point(data=GroupB, aes(y=MeanSeptumB9, x=DiameterB9), color='white')+

geom_point(data=GroupB, aes(y=MeanSeptumB10, x=DiameterB10), color='white')+

geom_point(data=GroupB, aes(y=MeanSeptumB11, x=DiameterB11), color='white')+

geom_point(data=GroupB, aes(y=MeanSeptumB12, x=DiameterB12), color='white')+

geom_point(data=GroupB, aes(y=MeanSeptumB13, x=DiameterB13), color='white')+

geom_point(data=GroupB, aes(y=MeanSeptumB14, x=DiameterB14), color='white')+

geom_point(data=GroupB, aes(y=MeanSeptumB15, x=DiameterB15), color='white')+

geom_point(data=GroupB, aes(y=MeanSeptumB16, x=DiameterB16), color='white')+

geom_point(data=GroupB, aes(y=MeanSeptumB17, x=DiameterB17), color='white')+

geom_point(data=GroupB, aes(y=MeanSeptumB18, x=DiameterB18), color='white')+

geom_point(data=GroupB, aes(y=MeanSeptumB19, x=DiameterB19), color='white')+

geom_point(data=GroupB, aes(y=MeanSeptumB20, x=DiameterB20), color='white')+

geom_point(data=GroupB, aes(y=MeanSeptumB21, x=DiameterB21), color='white')+

geom_point(data=GroupB, aes(y=MeanSeptumB22, x=DiameterB22), color='white')+

geom_point(data=GroupB, aes(y=MeanSeptumB23, x=DiameterB23), color='white')+

geom_point(data=GroupB, aes(y=MeanSeptumB24, x=DiameterB24), color='white')+

geom_point(data=GroupB, aes(y=MeanSeptumB25, x=DiameterB25), color='white')+

geom_abline(intercept = xxx, slope = xxx, col="red")+ #Lytos Cretaceous

theme_classic()

last_plot() + xlab("Septa thickness [cm]")

last_plot() + ylab("Diameter [cm]")

ggplot() +

geom_point(data=Group.A, aes(y=MeanSeptumA1, x=DiameterA1), color='white') +

geom_point(data=Group.A, aes(y=MeanSeptumA2, x=DiameterA2), color='white')+

geom_point(data=Group.A, aes(y=MeanSeptumA3, x=DiameterA3), color='white') +

geom_point(data=Group.A, aes(y=MeanSeptumA4, x=DiameterA4), color='white') +

geom_point(data=Group.A, aes(y=MeanSeptumA5, x=DiameterA5), color='white') +

geom_point(data=Group.A, aes(y=MeanSeptumA6, x=DiameterA6), color='white') +

geom_point(data=Group.A, aes(y=MeanSeptumA7, x=DiameterA7), color='white') +

geom_point(data=Group.A, aes(y=MeanSeptumA8, x=DiameterA8), color='white') +

geom_point(data=Group.B, aes(y=MeanSeptumB1, x=DiameterB1), color='white') +

geom_point(data=Group.B, aes(y=MeanSeptumB2, x=DiameterB2), color='white')+

geom_point(data=Group.B, aes(y=MeanSeptumB3, x=DiameterB3), color='white')+

geom_point(data=Group.B, aes(y=MeanSeptumB4, x=DiameterB4), color='white')+

geom_point(data=Group.B, aes(y=MeanSeptumB5, x=DiameterB5), color='white')+

geom_point(data=Group.B, aes(y=MeanSeptumB6, x=DiameterB6), color='white')+

geom_point(data=Group.B, aes(y=MeanSeptumB7, x=DiameterB7), color='white')+

geom_point(data=Group.B, aes(y=MeanSeptumB8, x=DiameterB8), color='white')+

geom_point(data=Group.B, aes(y=MeanSeptumB9, x=DiameterB9), color='white') +

geom_point(data=Group.B, aes(y=MeanSeptumB10, x=DiameterB10), color='white') +

geom_point(data=Group.B, aes(y=MeanSeptumB11, x=DiameterB11), color='white') +

geom_point(data=Group.B, aes(y=MeanSeptumB12, x=DiameterB12), color='white') +

geom_point(data=Group.B, aes(y=MeanSeptumB13, x=DiameterB13), color='white') +

geom_point(data=Group.B, aes(y=MeanSeptumB14, x=DiameterB14), color='white') +

geom_point(data=Group.B, aes(y=MeanSeptumB15, x=DiameterB15), color='white') +

geom_point(data=Group.B, aes(y=MeanSeptumB16, x=DiameterB16), color='white') +

geom_point(data=Group.B, aes(y=MeanSeptumB17, x=DiameterB17), color='white') +

geom_point(data=Group.B, aes(y=MeanSeptumB18, x=DiameterB18), color='white') +

geom_point(data=Group.B, aes(y=MeanSeptumB19, x=DiameterB19), color='white') +

geom_point(data=Group.B, aes(y=MeanSeptumB20, x=DiameterB20), color='white') +

geom_point(data=Group.B, aes(y=MeanSeptumB21, x=DiameterB21), color='white') +

geom_point(data=Group.B, aes(y=MeanSeptumB22, x=DiameterB22), color='white') +

geom_point(data=Group.B, aes(y=MeanSeptumB23, x=DiameterB23), color='white') +

geom_point(data=Group.B, aes(y=MeanSeptumB24, x=DiameterB24), color='white') +

geom_point(data=Group.B, aes(y=MeanSeptumB25, x=DiameterB25), color='white') +

geom_point(data=Group.C, aes(y=MeanSeptumC1, x=DiameterC1), color='white')+

geom_point(data=Group.D, aes(y=MeanSeptumD1, x=DiameterD1), color='white')+

geom_point(data=Group.E, aes(y=MeanSeptumE1, x=DiameterE1), color='white')+

geom_point(data=Group.E, aes(y=MeanSeptumE2, x=DiameterE2), color='white')+

geom_point(data=Group.E, aes(y=MeanSeptumE3, x=DiameterE3), color='white')+

geom_point(data=Group.E, aes(y=MeanSeptumE4, x=DiameterE4), color='white')+

geom_point(data=Group.E, aes(y=MeanSeptumE5, x=DiameterE5), color='white')+

geom_point(data=Group.F, aes(y=MeanSeptumF1, x=DiameterF1), color='white') +

geom_point(data=Group.F, aes(y=MeanSeptumF2, x=DiameterF2), color='white') +

geom_point(data=Group.F, aes(y=MeanSeptumF3, x=DiameterF3), color='white') +

geom_point(data=Group.G, aes(y=MeanSeptumG1, x=DiameterG1), color='white') +

geom_point(data=Group.H, aes(y=MeanSeptumH1, x=DiameterH1), color='white')+

geom_point(data=J1.und.J2, aes(y=xJ1, y=yJ1), color='white')+

geom_point(data=J1.und.J2, aes(y=xJ2, y=yJ2), color='white')+

geom_point(data=Group.K, aes(y=MeanSeptumK1, x=DiameterK1), color='white')+

geom_point(data=Group.L, aes(y=MeanSeptumL1, x=DiameterL1), color='white')+

geom_point(data=Group.M2, aes(y=MeanSeptumM1, x=DiameterM1), color='white')+

geom_point(data=Group.M2, aes(y=MeanSeptumM2, x=DiameterM2), color='white')+

geom_point(data=Group.M2, aes(y=MeanSeptumM3, x=DiameterM3), color='white')+

geom_point(data=Group.M2, aes(y=MeanSeptumM4, x=DiameterM4), color='white')+

geom_point(data=Group.N, aes(y=MeanSeptumN1, x=DiameterN1), color='white')+

theme_bw()+

geom_abline(intercept = xxx, slope = xxx, col="white")+ #*Arietites* sp.

geom_abline(intercept =xxx, slope = xxx, col="white")+ #Ammonitida

geom_abline(intercept = xxx, slope = xxx, col="red")+ ## Psiloceratidae

labs(title = "",

subtitle = "")

last_plot() + xlab("Septa Tickness [cm]")

last_plot() + ylab("Diameter [cm]")

last_plot() + theme_classic()

**Code Figure 11**

ggplot() +

geom_point(data=Group.A, aes(y=MeanSeptumA1, x=DiameterA1), color='navy', pch=22, bg="navy") +

geom_point(data=Group.A, aes(y=MeanSeptumA2, x=DiameterA2), color='navy', pch=22, bg="navy")+

geom_point(data=Group.A, aes(y=MeanSeptumA3, x=DiameterA3), color='navy', pch=22, bg="navy") +

geom_point(data=Group.A, aes(y=MeanSeptumA4, x=DiameterA4), color='navy', pch=22, bg="navy") +

geom_point(data=Group.A, aes(y=MeanSeptumA5, x=DiameterA5), color='navy', pch=22, bg="navy") +

geom_point(data=Group.A, aes(y=MeanSeptumA6, x=DiameterA6), color='navy', pch=22, bg="navy") +

geom_point(data=Group.A, aes(y=MeanSeptumA7, x=DiameterA7), color='navy', pch=22, bg="navy") +

geom_point(data=Group.A, aes(y=MeanSeptumA8, x=DiameterA8), color='navy', pch=22, bg="navy") +

geom_point(data=Group.B, aes(y=MeanSeptumB1, x=DiameterB1), color='black', pch=24, bg="black") +

geom_point(data=Group.B, aes(y=MeanSeptumB2, x=DiameterB2), color='black', pch=24, bg="black")+

geom_point(data=Group.B, aes(y=MeanSeptumB3, x=DiameterB3), color='black', pch=24, bg="black")+

geom_point(data=Group.B, aes(y=MeanSeptumB4, x=DiameterB4), color='black', pch=24, bg="black")+

geom_point(data=Group.B, aes(y=MeanSeptumB5, x=DiameterB5), color='black', pch=24, bg="black")+

geom_point(data=Group.B, aes(y=MeanSeptumB6, x=DiameterB6), color='black', pch=24, bg="black")+

geom_point(data=Group.B, aes(y=MeanSeptumB7, x=DiameterB7), color='black', pch=24, bg="black")+

geom_point(data=Group.B, aes(y=MeanSeptumB8, x=DiameterB8), color='black', pch=24, bg="black")+

geom_point(data=Group.B, aes(y=MeanSeptumB9, x=DiameterB9), color='black', pch=24, bg="black") +

geom_point(data=Group.B, aes(y=MeanSeptumB10, x=DiameterB10), color='black', pch=24, bg="black") +

geom_point(data=Group.B, aes(y=MeanSeptumB11, x=DiameterB11), color='black', pch=24, bg="black") +

geom_point(data=Group.B, aes(y=MeanSeptumB12, x=DiameterB12), color='black', pch=24, bg="black") +

geom_point(data=Group.B, aes(y=MeanSeptumB13, x=DiameterB13), color='black', pch=24, bg="black") +

geom_point(data=Group.B, aes(y=MeanSeptumB14, x=DiameterB14), color='black', pch=24, bg="black") +

geom_point(data=Group.B, aes(y=MeanSeptumB15, x=DiameterB15), color='black', pch=24, bg="black") +

geom_point(data=Group.B, aes(y=MeanSeptumB16, x=DiameterB16), color='black', pch=24, bg="black") +

geom_point(data=Group.B, aes(y=MeanSeptumB17, x=DiameterB17), color='black', pch=24, bg="black") +

geom_point(data=Group.B, aes(y=MeanSeptumB18, x=DiameterB18), color='black', pch=24, bg="black") +

geom_point(data=Group.B, aes(y=MeanSeptumB19, x=DiameterB19), color='black', pch=24, bg="black") +

geom_point(data=Group.B, aes(y=MeanSeptumB20, x=DiameterB20), color='black', pch=24, bg="black") +

geom_point(data=Group.B, aes(y=MeanSeptumB21, x=DiameterB21), color='black', pch=24, bg="black") +

geom_point(data=Group.B, aes(y=MeanSeptumB22, x=DiameterB22), color='black', pch=24, bg="black") +

geom_point(data=Group.B, aes(y=MeanSeptumB23, x=DiameterB23), color='black', pch=24, bg="black") +

geom_point(data=Group.B, aes(y=MeanSeptumB24, x=DiameterB24), color='black', pch=24, bg="black") +

geom_point(data=Group.B, aes(y=MeanSeptumB25, x=DiameterB25), color='deepskyblue') +

geom_point(data=Group.C, aes(y=MeanSeptumC1, x=DiameterC1), color='black', pch=24, bg="black")

geom_point(data=Group.D, aes(y=MeanSeptumD1, y=DiameterD1), color='black', pch=24, bg="black")+

geom_point(data=Group.E, aes(y=MeanSeptumE1, x=DiameterE1), color='orange')+

geom_point(data=Group.E, aes(y=MeanSeptumE2, x=DiameterE2), color='orange')+

geom_point(data=Group.E, aes(y=MeanSeptumE3, x=DiameterE3), color='orange')+

geom_point(data=Group.E, aes(y=MeanSeptumE4, x=DiameterE4), color='orange')+

geom_point(data=Group.E, aes(y=MeanSeptumE5, x=DiameterE5), color='orange')+

geom_point(data=Group.F, aes(y=MeanSeptumF1, x=DiameterF1), color='navy', pch=22, bg="navy") +

geom_point(data=Group.F, aes(x=MeanSeptumF2, y=DiameterF2), color='navy', pch=22, bg="navy") +

geom_point(data=Group.F, aes(x=MeanSeptumF3, y=DiameterF3), color='navy', pch=22, bg="navy") +

geom_point(data=Group.G, aes(x=MeanSeptumG1, y=DiameterG1), color='navy', pch=22, bg="navy") +

geom_point(data=Group.H, aes(y=MeanSeptumH1, x=DiameterH1), color='orange')+

geom_point(data=Group.J, aes(y=MeanSeptumJ1, x=DiameterJ1), color='deepskyblue')+

geom_point(data=Group.J, aes(y=MeanSeptumJ2, x=DiameterJ2), color='deepskyblue')+

geom_point(data=Group.J, aes(y=MeanSeptumJ3, x=DiameterJ3), color='deepskyblue')+

geom_point(data=Group.K, aes(y=MeanSeptumK1, x=DiameterK1), color='deepskyblue')+

geom_point(data=Group.L, aes(y=MeanSeptumL1, x=DiameterL1), color='deepskyblue')+

geom_point(data=Group.M, aes(y=MeanSeptumM1, x=DiameterM1), color='deepskyblue')+

geom_point(data=Group.M, aes(y=MeanSeptumM2, x=DiameterM2), color='deepskyblue')+

geom_point(data=Group.M, aes(y=MeanSeptumM3, x=DiameterM3), color='deepskyblue')+

geom_point(data=Group.M, aes(y=MeanSeptumM4, x=DiameterM4), color='deepskyblue')+

geom_point(data=Group.N, aes(y=MeanSeptumN1, x=DiameterN1), color='deepskyblue')+

geom_point(data=Group.O, aes(y=MeanSeptumO1, x=DiameterO1), color='orange')+

geom_point(data=Group.O, aes(y=MeanSeptumO2, x=DiameterO2), color='orange')+

theme_bw()+

geom_abline(intercept =xxx , slope =xxx, color='black')+ ## early Jurassic

geom_abline(intercept =xxx, slope = xxx, color='black')+ ## Triassic

geom_abline(intercept =xxx, slope = xxx, color='black')+ ## middle late Jurassic

geom_abline(intercept =xxx, slope = xxx, color='black')+ ## Cretaceous

labs(title = ,

subtitle = )

last_plot() + xlab("Septa thickness [cm]")

last_plot() + ylab("Diameter [cm]")

last_plot() + theme_classic()

**Code Figure 12**

install.packages("Kendall")

library(Kendall)

Slope<-data(0.0078, 0.0028, 0.0124, 0.0107, 0.004, 0.0037, 0.0041, 0.0024, 0.0004, 0.001, 0.0027, 0.0067, 0.0019, 0.0012, 0.0064, 0.0036, 0.0047, 0.0034, 0.0048, 0.0098, 0.0147, 0.0077, 0.0054, 0.0067, 0.0075)

Age<-c(105.5, 105.5, 105.5, 105.5, 106.75, 154.3, 168.8, 172.2, 172.2, 176.5, 178.4, 187.7,

195.05, 195.05, 195.05, 196.1, 200.3, 200.3, 222.8, 222.8, 222.8, 222.8, 222.8, 222.8, 222.8)

Kendall(Age, Slope)

plot(Age, Slope, col="black")

lines(lowess(time(slope, Age), Slope, Age), col="blue", lwd=2)

last_plot() + theme_classic()

**Code Figure 13**

slope<-data(124.89, 308.48, 78.83, 91.89, 188.35, 277.11, 239.38, 340.90, 322.27, 389.77, 236.30, 213.72, 417.20, 943.90, 102.53, 312.85, 312.61, 320.93, 90.78, 90.74, 87.31, 122.82, 153.15, 142.97, 133.11)

Age<-c(105.5, 105.5, 105.5, 105.5, 106.75, 154.3, 168.8, 172.2, 172.2, 176.5, 178.4, 187.7, 195.05, 195.05, 195.05, 196.1, 200.3, 200.3, 222.8, 226.6, 226.6, 226.6, 226.6, 226.6, 226.6)

plot(Age,slope, type="l")

**Statistical tests**

**Mann-Kendall test**

install.packages("Kendall")

library(Kendall)

Slope<-data(0.0078, 0.0028, 0.0124, 0.0107, 0.004, 0.0037, 0.0041, 0.0024, 0.0004, 0.001, 0.0027, 0.0067, 0.0019, 0.0012, 0.0064, 0.0036, 0.0047, 0.0034, 0.0048, 0.0098, 0.0147, 0.0077, 0.0054, 0.0067, 0.0075)

Age<-c(105.5, 105.5, 105.5, 105.5, 106.75, 154.3, 168.8, 172.2, 172.2, 176.5, 178.4, 187.7,

195.05, 195.05, 195.05, 196.1, 200.3, 200.3, 222.8, 222.8, 222.8, 222.8, 222.8, 222.8, 222.8)

Kendall(Age, Slope)

plot(Age, Slope, col="black")

lines(lowess(time(slope, Age), Slope, Age), col="blue", lwd=2)

last_plot() + theme_classic()

**Shapiro Wilk test**

Age<-c(105.5, 105.5, 105.5, 105.5, 106.75, 154.3, 168.8, 172.2, 172.2, 176.5, 178.4, 187.7,

195.05, 195.05, 195.05, 196.1, 200.3, 200.3, 222.8, 222.8, 222.8, 222.8, 222.8, 222.8, 222.8)

Slope<-c(0.0078, 0.0028, 0.0124, 0.0107, 0.004, 0.0037, 0.0041, 0.0024, 0.0004, 0.001, 0.0027, 0.0067, 0.0019, 0.0012, 0.0064, 0.0036, 0.0047, 0.0034, 0.0048, 0.0098, 0.0147, 0.0077, 0.0054, 0.0067, 0.0075)

co2<-c(1033.87, 1033.87,1033.87, 1033.87, 1033.87, 693.66, 875.22, 963.28, 963.28, 963.28, 963.28, 645.87, 870.46, 870.46, 870.46, 870.46, 870.46, 870.46, 1216.61, 1216.61, 1216.61, 1216.61, 1216.61, 1216.61, 1216.61)

mg/ca_ratio<-c(0.6, 0.6, 0.6, 0.6, 0.6, 1.45, 1.45, 1.51, 1.51, 1.52, 1.53, 1.9, 2.4, 2.4, 2.4, 2.45, 2.7, 2.7, 2.7, 2.6, 2.6, 2.6, 2.6, 2.6, 2.6)

ca2.conc<-c(5.15, 5.15, 5.15, 5.15, 5.15, 3.5, 3.5, 3.3, 3.3, 3.25, 3.2, 2.9, 2.5, 2.5, 2.5, 2.45, 2.3, 2.3, 2.2, 2.3, 2.3, 2.3, 2.3, 2.3, 2.3)

pH<-c(7.55, 7.55, 7.55, 7.55, 7.55, 7.62, 7.62, 7.62, 7.62, 7.6, 7.57, 7.54, 7.52, 7.52, 7.52, 7.52, 7.58, 7.58, 7.7, 7.73, 7.73, 7.73, 7.73, 7.73, 7.73)

Latitude_SN<-c(40, 40, 40, 40, 40, 20, 45, 50, 50, 50, 50, 50, 50, 50, 45, 50, 50, 45, 45, 25, 25, 25, 40, 25, 25)

shapiro.test(Slope)

shapiro.test(co2)

shapiro.test(Mg.Ca.ratio)

shapiro.test(Ca.conc)

shapiro.test(pH)

shapiro.test(Latitude)

shapiro.test(temp)

**Fligner Killeen test**

AbioticFactors <- read.csv("~/Desktop/AbioticFactors.csv", header=TRUE, sep=";")

View(AbioticFactors)

names(AbioticFactors)

[1] "Species" "Age.midpoint" "Septal.slope.factor" "CO2..Witkowski."

[5] "CO2..ppm." "Mg.Ca.ratio" "X.Ca2.." "pH"

[9] "Palaeolatitutde" "Latitude.SN"

install.packages("car")

library(car)

install.packages("carData")

library(carData)

fligner.test(Septal.slope.factor ~ Age.midpoint, data=AbioticFactors)

fligner.test(Septal.slope.factor ~ pH, data=AbioticFactors)

fligner.test(Septal.slope.factor ~ X.Ca2.., data=AbioticFactors)

fligner.test(Septal.slope.factor ~ Mg.Ca.ratio, data=AbioticFactors)

fligner.test(Septal.slope.factor ~ CO2..Witkowski., data=AbioticFactors)

fligner.test(Age.midpoint ~ CO2..Witkowski., data=AbioticFactors)

fligner.test(Age.midpoint ~ pH, data=AbioticFactors)

fligner.test(Age.midpoint ~ Mg.Ca.ratio, data=AbioticFactors)

fligner.test(Age.midpoint ~ X.Ca2.., data=AbioticFactors)

**Spearman rank test - Generalized differencing of time series**

source("http://www.graemetlloyd.com/pubdata/functions_2.r")

div1<-c(0.0078, 0.0028, 0.0124, 0.0107, 0.004, 0.0037, 0.0041, 0.0024, 0.0004, 0.001, 0.0027, 0.0067, 0.0019, 0.0012, 0.0064, 0.0036, 0.0047, 0.0034, 0.0048, 0.0098, 0.0147, 0.0077, 0.0054, 0.0067, 0.0075) #Slope

proxy1<-c(1033.87, 1033.87, 1033.87, 1033.87, 1033.87, 693.66, 875.22, 963.28, 963.28, 963.28, 963.28, 645.87, 870.46, 870.46, 870.46, 870.46, 870.46, 870.46, 1216.61, 1216.61, 1216.61, 1216.61, 1216.61, 1216.61, 1216.61) #CO2 Wotkowski

time<-c(105.5, 105.5, 105.5, 105.5, 106.75, 154.3, 168.8, 172.2, 172.2, 176.5, 178.4, 187.7,

195.05, 195.05, 195.05, 196.1, 200.3, 200.3, 222.8, 222.8, 222.8, 222.8, 222.8, 222.8, 222.8)

gd.div1<-gen.diff(div1,time)

gd.proxy1<-gen.diff(proxy1,time)

cor.test(gd.div1,gd.proxy1,method="spearman")

div1<-c(0.0078, 0.0028, 0.0124, 0.0107, 0.004, 0.0037, 0.0041, 0.0024, 0.0004, 0.001, 0.0027, 0.0067, 0.0019, 0.0012, 0.0064, 0.0036, 0.0047, 0.0034, 0.0048, 0.0098, 0.0147, 0.0077, 0.0054, 0.0067, 0.0075) #Slope

proxy1.1<-c(1650, 1650, 1650, 1650, 1700, 2430, 2430, 2200, 2200, 1950, 1800, 1650, 1550, 1550, 1550, 1500, 1100, 1100, 700, 750, 750, 750, 750, 750, 750) #CO2 Berner

time<-c(105.5, 105.5, 105.5, 105.5, 106.75, 154.3, 168.8, 172.2, 172.2, 176.5, 178.4, 187.7,

195.05, 195.05, 195.05, 196.1, 200.3, 200.3, 222.8, 222.8, 222.8, 222.8, 222.8, 222.8, 222.8)

gd.div1<-gen.diff(div1,time)

gd.proxy1.1<-gen.diff(proxy1.1,time)

cor.test(gd.div1,gd.proxy1.1,method="spearman")

div1<-c(0.0078, 0.0028, 0.0124, 0.0107, 0.004, 0.0037, 0.0041, 0.0024, 0.0004, 0.001, 0.0027, 0.0067, 0.0019, 0.0012, 0.0064, 0.0036, 0.0047, 0.0034, 0.0048, 0.0098, 0.0147, 0.0077, 0.0054, 0.0067, 0.0075)#Slope

proxy2<-c(40, 40, 40, 40, 40, 20, 45, 50, 50, 50, 50, 50, 50, 50, 45, 50, 50, 45, 45, 25, 25, 25, 40, 25, 25) #latitude

time<-c(105.5, 105.5, 105.5, 105.5, 106.75, 154.3, 168.8, 172.2, 172.2, 176.5, 178.4, 187.7,

195.05, 195.05, 195.05, 196.1, 200.3, 200.3, 222.8, 222.8, 222.8, 222.8, 222.8, 222.8, 222.8)

gd.div1<-gen.diff(div1,time)

gd.proxy2<-gen.diff(proxy2,time)

cor.test(gd.div1,gd.proxy2,method="spearman")

div1<-c(0.0078, 0.0028, 0.0124, 0.0107, 0.004, 0.0037, 0.0041, 0.0024, 0.0004, 0.001, 0.0027, 0.0067, 0.0019, 0.0012, 0.0064, 0.0036, 0.0047, 0.0034, 0.0048, 0.0098, 0.0147, 0.0077, 0.0054, 0.0067, 0.0075) #Slope

proxy3<-c(0.6, 0.6, 0.6, 0.6, 0.6, 1.45, 1.45, 1.51, 1.51, 1.52, 1.53, 1.9, 2.4, 2.4, 2.4,

2.45, 2.7, 2.7, 2.7, 2.6, 2.6, 2.6, 2.6, 2.6, 2.6) #mg/ca ratio

time<-c(105.5, 105.5, 105.5, 105.5, 106.75, 154.3, 168.8, 172.2, 172.2, 176.5, 178.4, 187.7,

195.05, 195.05, 195.05, 196.1, 200.3, 200.3, 222.8, 222.8, 222.8, 222.8, 222.8, 222.8, 222.8)

gd.div1<-gen.diff(div1,time)

gd.proxy3<-gen.diff(proxy3,time)

cor.test(gd.div1,gd.proxy3,method="spearman")

div1<-c(0.0078, 0.0028, 0.0124, 0.0107, 0.004, 0.0037, 0.0041, 0.0024, 0.0004, 0.001, 0.0027, 0.0067, 0.0019, 0.0012, 0.0064, 0.0036, 0.0047, 0.0034, 0.0048, 0.0098, 0.0147, 0.0077, 0.0054, 0.0067, 0.0075)#Slope

proxy3<-c(7.55, 7.55, 7.55, 7.55, 7.55, 7.62, 7.62, 7.62, 7.62, 7.6, 7.57, 7.54, 7.52, 7.52, 7.52, 7.52, 7.58, 7.58, 7.7, 7.73, 7.73, 7.73, 7.73, 7.73, 7.73)#pH

time<-c(105.5, 105.5, 105.5, 105.5, 106.75, 154.3, 168.8, 172.2, 172.2, 176.5, 178.4, 187.7, 195.05, 195.05, 195.05, 196.1, 200.3, 200.3, 222.8, 222.8, 222.8, 222.8, 222.8, 222.8, 222.8)

gd.div1<-gen.diff(div1,time)

gd.proxy3<-gen.diff(proxy3,time)

cor.test(gd.div1,gd.proxy3,method="spearman")
